# Therapeutic implications for localized prostate cancer by multiomics analyses of the ageing microenvironment landscape

**DOI:** 10.7150/ijbs.85209

**Published:** 2023-07-31

**Authors:** Chengpeng Gui, Jinhuan Wei, Chengqiang Mo, Yanping Liang, Junjie Cen, Yuhang Chen, Daohu Wang, Junhang Luo

**Affiliations:** 1 Department of Urology, First Affiliated Hospital, Sun Yat-sen University, Guangzhou, Guangdong, China.; 2 Department of Medical Biophysics, University of Toronto, Toronto, Ontario, Canada.; 3 Department of Laboratory Medicine, First Affiliated Hospital, Sun Yat-sen University, Guangzhou, Guangdong, China.; 4 Institute of Precision Medicine, First Affiliated Hospital, Sun Yat-sen University, Guangzhou, Guangdong, China.

**Keywords:** tumor ageing microenvironment, antiandrogen therapy, immunotherapy, spatial transcriptomics, prostate cancer

## Abstract

**Background:** Numerous studies have substantiated the association between aging and the progression of malignant tumors in humans, notably prostate cancer (PCa). Nevertheless, to the best of our knowledge, no studies have comprehensively elucidated the intricate characteristics of the aging microenvironment (AME) in PCa.

**Methods:** AME regulatory patterns were determined using the NMF algorithm. Then an ageing microenvironment index (AMI) was constructed, with excellent prognostic and immunotherapy prediction ability, and its' clinical relevance was surveyed through spatial transcriptomics. Further, the drug response was analysed using the Genomics of Drug Sensitivity in Cancer (GDSC), the Connectivity Map (CMap) and CellMiner database for patients with PCa. Finally, the AME was studied using in vitro and vivo experiments.

**Results:** Three different AME regulatory patterns were identified across 813 PCa patients, associated with distinct clinical prognosis and physiological pathways. Based on the AMI, patients with PCa were divided into the high-score and low-score subsets. Higher AMI score was significantly infiltrated with more immune cells, higher rate of biochemical recurrence (BCR) and worse response to immunotherapy, antiandrogen therapy and chemotherapy in PCa. In addition, we found that the combination of bicalutamide and embelin was capable of suppressing tumor growth of PCa. Besides, as the main components of AMI, *COL1A1* and* BGLAP* act as oncogenes and were verified via in vivo and in vitro experiments.

**Conclusions:** AME regulation is significantly associated with the diversity and complexity of TME. Quantitative evaluation of the AME regulatory patterns may provide promising novel molecular markers for individualised therapy in PCa.

## Introduction

According to the latest statistical data of cancer, prostate cancer (PCa) has exceeded lung cancer to have the highest incidence in the malignant tumor among men, and it has the second-highest mortality rate following lung cancer^1^. For localized PCa, radical prostatectomy and radiotherapy are recommended therapeutic strategies^2^. These treatments are capable of controlling the tumor to a great degree, but still 20-25% of the patients experience biochemical recurrence (BCR)^3^-^5^. The disease may progress clinically within 5-8 years if patients with BCR are not secondarily treated, among whom 32-45% may die of PCa within 15 years^6^. The underlying mechanisms of widespread BCR in PCa should be elucidated to expand benefits of chemotherapy, antiandrogenic therapy and immunotherapy for more patients.

Ageing is a time-dependent irreversible process characterized by slowly progressive degeneration of most physiological functions, and identified as a key threat for tumor-related diseases^7^. Cellular senescence is a permanent cell growth arrest, resulting in inhibition of the uncontrolled proliferation, migration, invasion and metastasis of tumor-prone cells^8^. However, effects of cellular senescence on tumors are extremely complicated. On the one hand, ageing-related genes (ARGs) suppress tumors by facilitating the senescence of tumor cells, while on the other hand, ARGs promote tumor growth, invasion, progression and metastasis of cancer^9^. Lately, the application of ARGs as diagnostic molecular biomarkers and prognostic indicators has caught the attention of cancer researchers^10^. However, the biological functions and the prognostic role of ARGs in PCa remain unclear, and the association between the ageing microenvironment and PCa progression has not been reported.

Herein, this study is the first to depict the cancerous ageing microenvironment landscape, including its molecular characteristics, tumor immunity patterns and clinical correlation. Our study highlights the significance of the ageing microenvironment in the pathogenesis of cancer and formation of the tumor immune microenvironment (TIME) and set a theoretical basis for guiding therapeutic strategies for patients with PCa.

## Materials and Methods

### Ethics Statement

The study received approval from the Institutional Ethics Committee of Sun Yat-sen University First Affiliated Hospital and the Institutional Animal Care and Use Committee of Sun Yat-sen University. The cell experimental protocols were approved by the Review Board of Sun Yat-sen University First Affiliated Hospital. All methods adhered to the relevant guidelines, regulations, and the declaration of Helsinki, including the ARRIVE guidelines. Informed consent was waived by the Medical Ethics Committee, as the archival samples (IHC analysis of *COL1A1* and *BGLAP*) were retrospectively collected from 78 pairs of tumor tissues and their corresponding normal tissues after radical prostatectomy. The data were collected and analyzed anonymously.

### Data acquisition and preparation

Transcriptomic data and related clinicopathological information from patients with PCa were derived from the TCGA, GEO, DKFZ databases and the MSKCC cohort. After removing cases with missing follow-up data, 813 cases (495 from TCGA, 96 from GSE54460, 82 from DKFZ and 140 from MSKCC) with tumor samples and clinicopathologic data were eventually included in the study (**Table 1**). In addition, the data of 52 normal tissues of the prostate were retrieved from TCGA database. 311 human ARGs were obtained from the Human Ageing Genomic Resources and Molecular Signatures Database (MSigDB), listed in **Table S1**. The ComBat method in the R package “SVA” was applied to eliminate the batch effects of transcriptomic data across different datasets^11^, ^12^ (**Figure S1**). Genomic mutation data (i.e. somatic mutations and copy number variations [CNVs]) of the TCGA-PRAD cohort were retrieved from the UCSC Xena website and the study by Davoli^13^. The CNV landscape of 36 AME regulators in human chromosomes was plotted through the R package “Rcircos”.

### Selection of AME regulators and consensus molecular clustering using NMF

The "limma" R package was used to identify BCR-related genes differentially expressed between BCR-positive and BCR-negative PCa tissues (FDR < 0.05, log2 fold change > 1). Univariate Cox analysis determined bRFS-related ARGs. The overlapping prognostic ARGs (n=36) in TCGA were selected as key genes to build a protein interaction network using the STRING database, and an expression correlation network of these key genes was also constructed. Consensus clustering with the "NMF" R package (Brunet algorithm, 200 runs) discovered distinct AME patterns based on 36 key genes in a meta-cohort (n=813).

### GSVA analysis and functional annotation

We utilized the "GSVA" R package^14^ to conduct gene set variation analysis (GSVA) in order to explore differences in biological processes across various AME regulatory patterns. Metascape was used to assess the functions of the 36 AME regulators and their co-expression genes, considering significance as p-value < 0.01, enrichment score > 1.5, and a minimum count of 3. Additionally, gene set enrichment analysis (GSEA), Kyoto Encyclopedia of Genes and Genomes (KEGG), and Gene Ontology (GO) analyses were performed to examine distinct signal pathways and molecular mechanisms between different patient groups. Significantly enriched genes were defined as having an FDR < 0.05 after conducting 1,000 permutations.

### Establishment of an immune landscape for distinct AME phenotypes

The relationship between AME regulators and immune phenotypes was examined based on three aspects. First, four clusters of metagenes representing different responses of immune and inflammation were selected. Second, immune and stromal scores were calculated by the Estimation of Stromal and Immune cells in Malignant tumor tissues using Expression data (ESTIMATE)^15^ and Microenvironment Cell Populations-counter (MCP-counter) methods^16^. We conducted a comprehensive investigation of the overall immune landscape, encompassing immune-cell infiltration and immune function, utilizing the single sample gene set enrichment analysis (ssGSEA) algorithm. The ssGSEA method was employed to assess the abundance of 17 immune-cell infiltration and 13 immune-function levels. The third aspect was immune checkpoint profiling, including PD-1 and PD-L1.

### Construction and validation of an ageing microenvironment index

The analysis emphasized the importance of AME, leading to the construction of a survival prediction signature using the "glmnet" R package in the training set, following a previously established method^17^. This resulted in the development of an ageing microenvironment index (AMI) formula for survival prediction. The prognostic model was evaluated and validated in four cohorts (TCGA, MSKCC, GSE54460, and DKFZ) using Kaplan-Meier (K-M) and receiver operating characteristic (ROC) analyses. Principal component analysis (PCA) was performed using the 'vegan' and 'stats' packages to examine the distribution of AMI across different subgroups. Cox regression analyses, including univariate and multivariate analyses, assessed the AMI as an independent prognostic factor for PCa patients. The correlation between AMI and clinical features was visualized through a heat map, and subgroup analyses explored the correlation between AMI and significant clinical features. Overall, this comprehensive approach demonstrated the significance of AMI in predicting PCa patient outcomes.

### Spatial transcriptomics data processing

The trimmed data (three prostate cancer patients and one normal human prostate) was downloaded from 10X Genomics website and mapped to the GRCH38 v93 genome assembly. We obtained the gene-spot matrices and then used the “Seurat” package (versions 3.0.0/3.1.3) to analyze the matrices. For each patient's data, spots were eliminated with minimum detected gene count less than 200 genes. And genes were selected when minimum read count exceeded 10 and expression spots exceeded 2. We used the AddModuleScore function with default parameters in Seurat (version 3.1.3) to calculate the signature scoring. Spatial feature expression plots were demonstrated with the SpatialFeaturePlot function in Seurat and the “STUtility” R package (version 1.0.0).

### Construction of a nomogram integrated with AMI and clinical factors

To develop a scoring system capable of predicting the 1-, 3- and 5-year bRFS of patients with PCa, we established a nomogram combining the AME signature and other clinical characteristics in the TCGA cohort. AUC values and calibration plots were generated to examine the prediction accuracy. Decision curve analysis (DCA) was performed to evaluate the clinical practicability of the nomogram.

### Predicting the effect of antiandrogenic therapy

Data regarding the response of PCa patients to bicalutamide, embelin and docetaxel in the four cohorts (TCGA, MSKCC, GSE54460 and DKFZ) were retrieved from the GDSC database by the R package “pRRophetic”^18^. The CMap database (https://portals.broadinstitute.org/cmap/) was utilized to explore potential drugs. P-values < 0.05 denoted statistical significance. Subsequently, the 3D structure diagrams of the candidate drugs were generated by PubChem (https://pubchem.ncbi.nlm.nih.gov/). We used CellMiner to screen for drugs targeting important genes to develop new treatment strategies for patients with PCa. Finally, an in-vivo efficacy assessment of drug-loaded micelles in xenografts was performed to validate the prediction (details in **Supplementary appendix: Supplementary methods**).

### Quantification of immune response prediction using immunophenoscore and TIDE and validating in two immunotherapy cohorts

Immunophenoscore (IPS) is a widely used index for treatment response prediction of anti-PD-1 and anti-CTLA-4 agents^19^. The tumor Immune Dysfunction and Exclusion (TIDE) algorithm was used to exhibit mechanisms of tumor immune escape^20^. Tumor cells with higher TIDE scores were tend to induce immune escape, leading to an unfavorable response to ICI treatment. Lastly, we stated the difference of immunotherapy response between AMI-high and AMI-low subgroups in GSE78220 and IMvigor cohort.

### Fitness gene analysis

Fitness genes were selected from a previous study^21^ and were defined as genes required for cell growth or viability in cancer cell lines. In addition, they were used to validate the essential role of *COL1A1* and *BGLAP* in cancer cells. DepMap Portal data was used to further validated the essential roles of *COL1A1* and *BGLAP* in cancer cells (https://depmap.org/portal/). And *COL1A1* and* BGLAP* act as oncogenes and were verified via in vivo and in vitro experiments (details in **Supplementary appendix: Supplementary methods**).

### Statistical analysis

Statistical analyses were conducted using Rstudio 4.2.2, SPSS 19.0 or GraphPad Prism 9.0 software. All data and error bars are presented as the mean ± SDs from at least three independent experiments. A Wilcox test was used to compare differences between two groups. Survival curves were generated using the Kaplan-Meier method and compared using the log-rank test. To investigate the correlation between two independent groups, the Pearson's Chi-square test was used. The receiver operating characteristic curve (ROC) was drawn to evaluate the predictive ability for BCR. The indicated P values (*P < 0.05, **P < 0.01 and ***P < 0.001) were considered statistically significant. For all of the experiments shown, n is indicated in the figure legends. The values represent the mean. The error bars, if shown, represent the s.d. (as indicated in the figure legends).

## Results

### Landscape of genetic variations, expression patterns and therapeutic potential of AME regulators in PCa

**Figure 1:**
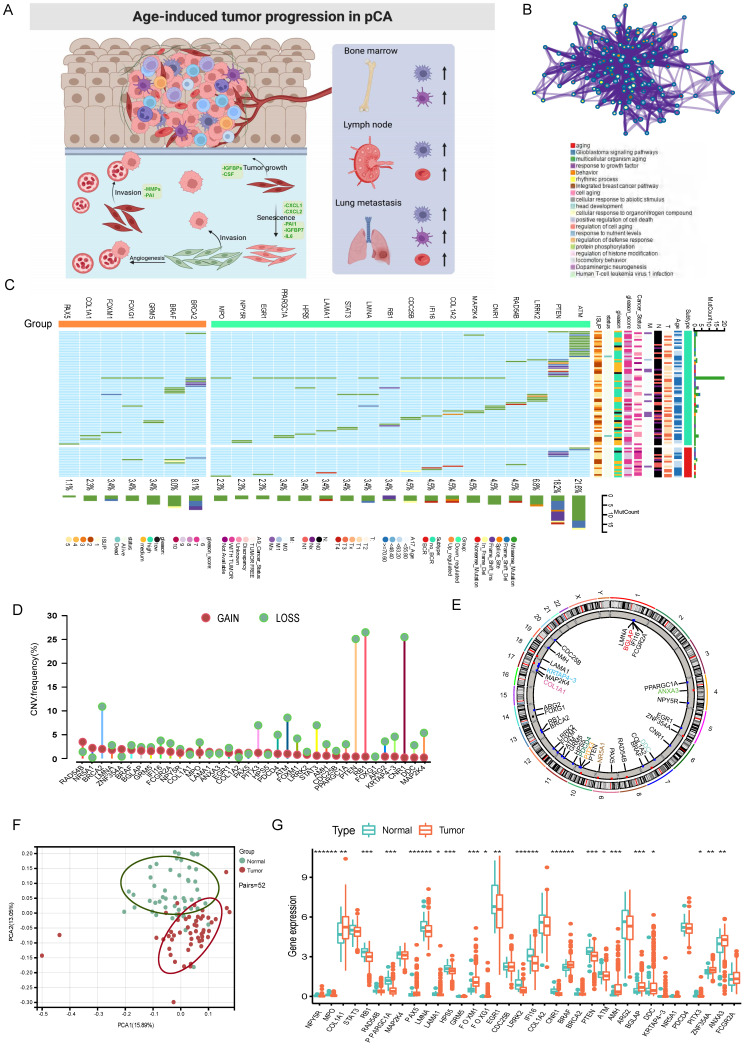
The panorama of genetic variation, expression patterns, and therapeutic potential of AME regulators in prostate cancer. (A) Summary of the current knowledge about the dynamic reversible process of aged tumor microenvironment in cancer progression; (B) Metascape enrichment network visualization showed the intra-cluster and inter-cluster similarities of enriched terms, up to 20 terms per cluster. Cluster annotations were shown in the color code; (C) The mutation frequency of top 25 AME regulators in 483 patients with prostate cancer from the TCGA Cohort. Each column corresponds to an individual case. The TMB is displayed as the right bar plot. The panel on the bottom shows the mutation frequency and proportion of each variant type for each regulator. The stacked bar plot on the left displays the fraction of conversions in each patient. (D) The copy number variation frequency of 36 AME regulators in 495 PCa tissues from TCGA-PRAD. Green dot, the deletion frequency; Red dot, The amplifcation frequency. (E) The location of CNV alteration of the AME regulators on 23 chromosomes using data from TCGA. (F) Principal component analysis for the expression profiles of 36 AME regulators to distinguish paired prostate cancer samples from normal prostate samples in TCGA cohort. There is no intersection between the two subgroups, indicating the prostate cancer samples and normal prostate samples were well distinguished based on the expression profiles of AME regulators. PCa samples were marked with red and normal prostate samples were marked with green. (G) The expression detail of 36 AME regulators between normal prostate tissues and prostate cancer tissues from TCGA cohort. Green box, normal prostate samples; orange box, prostate cancer samples.

We identified 36 AME regulators, comprising 18 bRFS-positive and 18 bRFS-negative regulators (**Table S2**), which impact cancer tumorigenesis and progression (**Fig. 1A**). Analysis using the Metascape database revealed enrichment in terms related to ageing, response to growth factor, protein phosphorylation, regulation of histone modification, and human T-cell leukemia virus 1 infection (**Fig. 1B**). A protein-protein interaction (PPI) network demonstrated strong expression correlation among these genes (**Fig. S2C**), and correlation analysis further supported their transcriptional level correlation (**Fig. S2E**).

Somatic mutations of the top 25 AME regulators were examined in 483 PCa samples, revealing a mutation frequency of 17.39% (84 samples) (**Fig. 1C**). Downregulated regulators showed higher mutation frequencies, notably *ATM*, *PTEN*, and *LRRK2*. Co-occurring mutations were observed between *COL1A1* and *PPARGC1A*, as well as between *FOXM1* and *STAT3* (**Fig. S2D**).

Furthermore, CNVs of these regulators were investigated in 496 PCa samples from the TCGA cohort, showing universal CNV patterns (**Fig. 1D**). Most downregulated regulators (18/36) showed widespread copy number amplification, while upregulated regulators had copy number deletion. Chromosomal CNV patterns are depicted in **Fig. 1E**. Due to the common genetic alterations of AME regulators in PCa, it is important to assess whether these changes affect their expression. PCA analysis demonstrated distinct distribution patterns of AME regulators between PCa samples and normal prostate tissues (**Fig. 1F**). The expression of most regulators showed significant differences in PCa, suggesting enriched AME regulation. Notably, the expression of *RB1* and *CNR1*, known to be significantly low in multiple solid tumors^22^, ^23^, may be influenced by CNVs (**Fig. 1G**).

### Identification of tumor ageing microenvironment patterns mediated by 36 AME regulators

**Figure 2:**
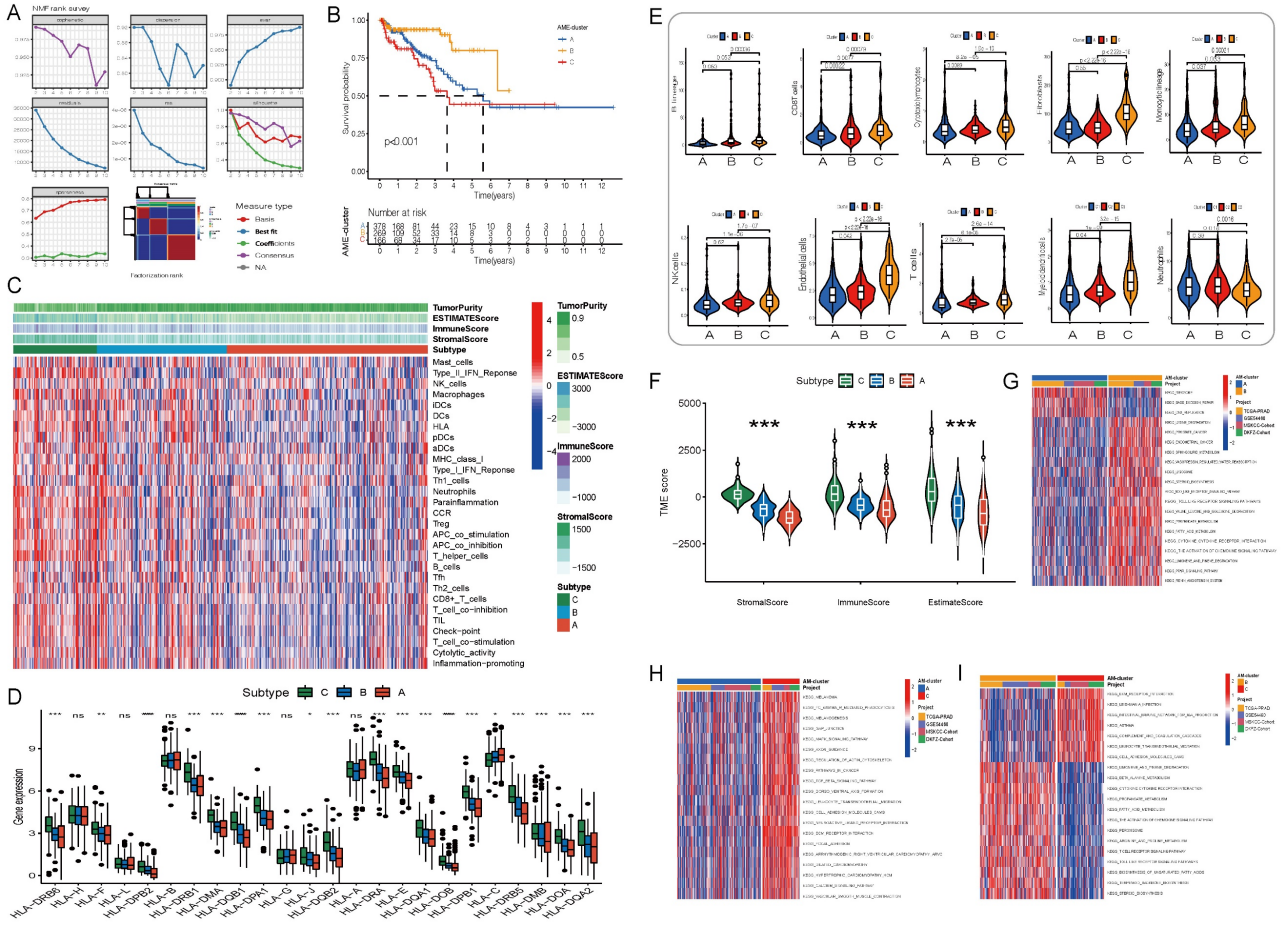
Initial screening of potential prognostic AME regulators and establish the AME phenotypes. (A) Consensus molecular clustering of thirty-six AME regulators by NMF. (B) Kaplan-Meier curves of biochemical relapse-free survival (bRFS) for 813 PCa patients in meta cohort with different AME clusters. The numbers of patients in AME-A, AME-B, and AME-C phenotypes are 378, 269, and 166, respectively (Log-rank test). (C) Unsupervised clustering of 36 AME regulators in the meta PCa cohort. Estimate score, Immune score, Stromal score, Tumor Purity, as well as the AME cluster, is shown in annotations above. Red represented the high expression of regulators and blue represented the low expression. (D) Gene expression of HLA gene sets between two distinct subgroups. (E) The fraction of tumor-infiltrating lymphocyte cells in three AME clusters using the MCP counter algorithm. Within each group, the scattered dots represented TME cell expression values. The thick line represented the median value. The bottom and top of the boxes were the 25th and 75th percentiles (interquartile range). The statistical difference of three gene clusters was compared through the Kruskal-Wallis H test. *P < 0.05; **P < 0.01; ***P < 0.001. (F) Estimate score between High AMI and Low AMI group. (G-I) GSVA enrichment analysis showing the activation states of biological pathways in distinct AME regulation patterns. The heatmap was used to visualize these biological processes, and red represented activated pathways and blue represented inhibited pathways. The prostate cancer cohorts were used as sample annotations. (G) AME cluster A vs AME cluster B; (H) AME cluster A vs AME cluster C; (I) AME cluster B vs AME cluster C.

A comprehensive landscape of the interactions among the 36 AME regulators and their prognostic value in PCa were demonstrated in an AME regulatory network in the meta-cohort (**Fig. S2E**). The results indicated that the cross-talk among the regulators may play a critical role to form different AME regulatory patterns and are involved in carcinogenesis and tumor progression. Based on this hypothesis, three distinct AME patterns were identified using the NMF algorithm, including 378 cases in cluster AME-A, 269 cases in AME-B and 166 cases in AME-C (**Fig. 2A-B**). And cases in AME-B exhibited a marked survival advantage, whereas the prognosis of cases in AME-C was worst among the three clusters (P < 0.001, log-rank test).

### Characteristics of immune cell infiltration in distinct AME regulatory patterns in PCa

To examine biological behaviours among the three AME regulatory patterns, GSVA was performed. As shown in **Fig. 2G-I**, AME-C was significantly enriched in carcinogenic activation and stromal pathways such as ECM receptor interaction, transforming growth factor beta (TGF-β) signalling pathway, cell adhesion and MAPK signalling pathway. AME-B was markedly enriched in pathways involved in immune activation including the activation of chemokine signalling pathway, T cell receptor signalling pathway, Toll-like receptor signalling and cytokine-cytokine receptor interaction pathway, whereas AME-A was prominently enriched in pathways related to immune suppression.

To further ascertain the underlying biological behaviour of each AME regulatory pattern, 1577 AME phenotype-related DEGs were obtained using the “limma” R package (**Fig. S3A**). Then significantly enriched biological processes from GO enrichment analysis were summarised in **Fig. S3C**. These DEGs were enriched in biological processes associated with cancer-related pathways and immunity, which verified that AME regulators are important for immune regulation of the tumor microenvironment (**Fig. S3B-S3C**).

Furthermore, the MCP-counter and ESTIMATE methods were used to examine the distribution of immune and stromal cells based on the expression of AME regulators (**Fig. 2C-E, Fig. S3D**). Among all the patients, the estimated immune scores ranged from -1452.5 to 2989.1. Shown in **Fig. 2F**; **Fig. S3F**, the estimate, immune and stromal scores of the AME-C cluster were significantly higher than those of the other two clusters (P < 0.05, Mann-Whitney U-test). Differences in the distribution of scores among the three AME clusters were assessed, and the AME-C cluster was found to have lowest tumor purity. In addition, we investigated the relationship between HLA genes and the three AME clusters. As displayed in **Fig. 2D**, the expression of HLA genes was significantly higher in the AME-B and AME-C clusters than in the AME-A cluster (FDR < 0.05, Mann-Whitney U-test). In addition, the relationship between three distinct AME patterns and the suppression/activation state of immune function as well as the related chemokine/cytokine/inflammatory factor changes was added, as shown in the Figure S3E. The cluster C showed higher levels of aging-related chemokines/cytokines/inflammatory factors, consistent with previous findings.

Moreover, the analysis of TME cell infiltration revealed that the AME-C cluster was remarkably enriched with innate immune cell infiltration including natural killer (NK) cells, macrophages, T cells, CD8 T cells, mast cells, MDSCs and plasmacytoid dendritic cells (**Fig. 2C, 2E**). However, patients in the AME-C cluster did not exhibit a matched survival advantage (**Fig. 2B**). Previous research has revealed that tumors with immune-excluded phenotypes have abundant immune cells; however, these immune cells are restricted in the stroma encircling tumor cell nests instead of penetrating into the tumor parenchyma. In addition, the activation of stroma in TME suppresses T cells^24^. The results of GSVA suggested that the AME-C regulation pattern was markedly related to stromal activation. Therefore, we could speculate that stromal activation of the AME-C cluster may inhibit the anti-tumor effects of immune cells. Further analyses revealed that stromal activity was distinctly increased in the AME-C cluster, including the activation of epithelial-mesenchymal transition (EMT), TGF-β pathway and angiogenesis, thus verifying our speculation (**Fig. 2G-I**). It's known that PD-L1 is an acknowledged biomarker to predict the anti-PD-1/L1 therapy response^25^, thus we compared PD-1/L1 expression among different AME regulatory clusters and found an obvious upregulation in the AME-B cluster (**Fig. S3G**).

Based on the abovementioned analyses, we found that the three AME regulatory patterns had notably distinct TME cell infiltration phenotypes. AME-C was identified as an immune-excluded phenotype characterised by innate immune cell infiltration and stromal activation. AME-B was identified as an immune-inflamed phenotype characterised by adaptive immune cell infiltration and immune activation, and AME-A was identified as an immune-desert phenotype characterised by immune suppression (**Fig. 2G-I and Fig. 2C-E**).

### Clinical significance of quantitative AME regulators (AMI) in PCa

Given the association between AME regulators and immune-oncological features, we assessed their clinical significance in PCa. Because most regulators (22/36) exhibited clinical relevance (**Table S2**), we constructed an AME regulator-based signature (ageing microenvironment index [AMI]) for prediction. In the training set of TCGA cohort, 36 ARGs were subjected to LASSO regression analysis (**Fig. S4A**); 12 genes identified via LASSO were subsequently analysed by multivariate Cox regression to develop the risk signature (**Fig. S4A**). As a result, a risk signature containing eight ARGs was constructed based on 248 PCa cases in the training set of TCGA cohort (**Fig. S4B**). Specifically, the AMI formula was constructed based on a linear combination of the expression of 8 ARGs weighted with the regression coefficients of the multivariate Cox regression analysis. The formula is: AMI =*COL1A1* × 0.664883214 + *BGLAP* × 0.882893312 + *DDC* × 0.619482468 - *KRTAP4-3* × 6.72283363 - *NR5A1* × 12.2363945 - *PDCD4* × 1.092162892 - *PITX3* × 8.308315706 - *ANXA3* × 0.348239554.

**Figure 3:**
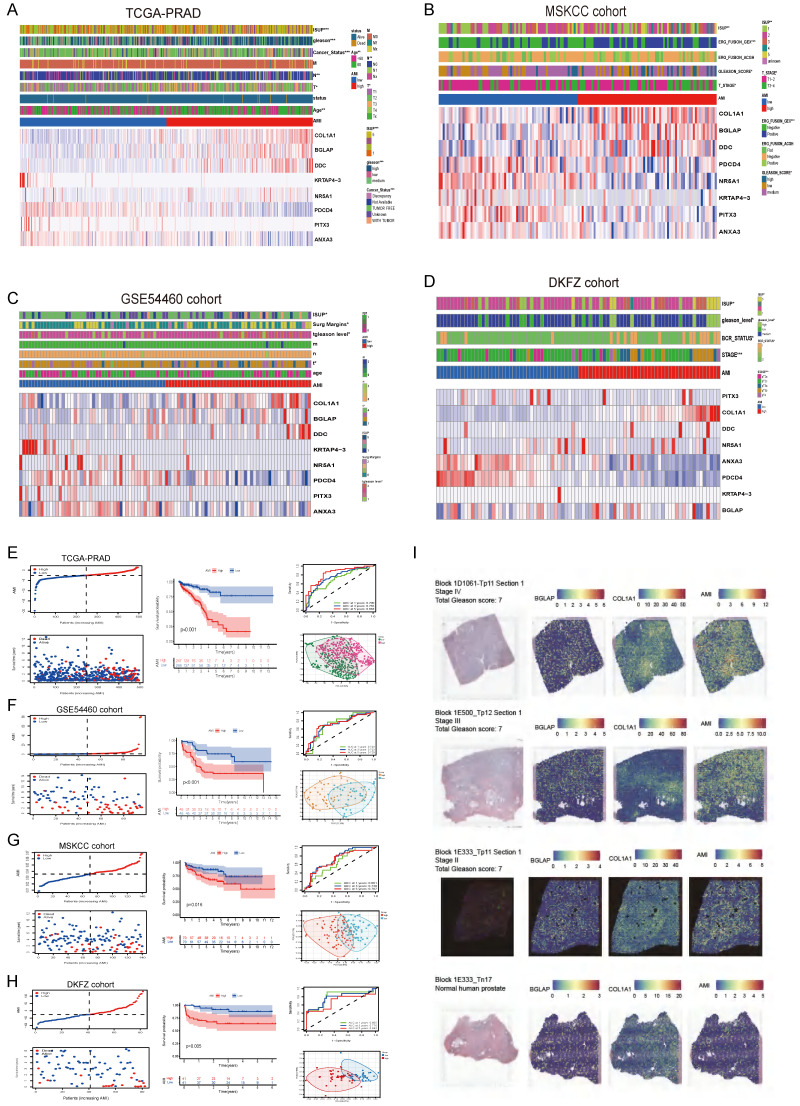
The relationship between prognosis and clinicopathological parameters in four independent cohorts (*p < 0.05; **p < 0.01; ***p < 0.001). (A-D) The distribution of AMI, biochemical recurrence status along with bRFS times of PCa patients and heatmaps of 8 key prognostic AME regulators. (E-H) Kaplan-Meier survival curves of bRFS and ROC analysis of the AME signature indicated that the signature has good bRFS predictive. And The two risk groups based on AMI were distinguished by PCA. (I) H&E, BGLAP, COL1A1 expression and AMI feature plots from three prostate cancer patients and one normal human prostate with data generated using the Visium ST platform.

Further, we analysed spatial transcriptomics data from three prostate cancer patients and one normal human prostate. We found that patients with higher stage had higher level of AMI (**Fig. 3I**). And more *COL1A1* and *BGLAP* are expressed in the tumor than in the normal prostate (**Fig. 3I**).

### Evaluation and validation of the prognostic significance of AMI

Using the median AMI as the cut-off value, patients were divided into the low- and high-AMI groups (**Fig. S4B-C, 3F-H**). The BCR status and follow-up time of patients with PCa were demonstrated in **Fig. S4B-C, 3F-H**. In addition, a heatmap demonstrating the expression profiles of the eight ARGs was plotted (**Fig. 3A-D; Fig. S4B-C**). Kaplan-Meier survival curves of the low- and high-AMI groups in the training set are shown in **Fig. S4B** (P < 0.001). The signature was evaluated using time-dependent ROC curves for predicting prognosis, with the AUC values for predicting 1-, 3- and 5-year OS in the training set being 0.793, 0.846 and 0.893, respectively (**Fig. S4B**).

To determine the applicability of AMI in clinical practice, we verified the repeatability and robustness of AMI using an internal validation set (**Fig. S4C; Fig. 3A,E**) and three independent cohorts (**Fig. 3F-H**). We found that the classifier significantly stratified patients with bRFS into the high- and low-AMI groups (**Fig. 3E-H; Fig. S4C, P < 0.05**). The AUC values of the prognostic model for predicting 5-year bRFS were 0.893, 0.783, 0.838, 0.729, 0.707 and 0.692 in the TCGA training cohort, TCGA testing cohort, whole TCGA cohort, GSE54460 cohort, MSKCC cohort and DKFZ cohort, respectively (**Fig. S4B-C; Fig. 3E-H**).

To further verify the prognostic value of the AME signature for diverse clinical and pathological characteristics, subgroup analysis was applied across the four PCa cohorts. AMI was verified in different clinical subgroups (**Fig. S5-S6**) and performed well in patients aged >60 years, those with higher ISUP levels or Gleason scores, those with T3-T4 stage disease and those with positive surgical margin, regardless of the cohort (**Fig. S5-S6**, P < 0.001). The incidence of BCR, the WHO ISUP classification grade, GS and T-staging were higher in the high-AMI group than in the low-AMI group. In addition, after adjusting for clinical and pathological characteristics, AMI was identified as an independent prognostic factor for patients with PCa (**Fig. S7A-F**). The results of PCA analyses revealed that different AMI subgroups exhibited distinct discrete tendency in the two-dimensional plane (**Fig. 3A-D**).

### Comparison of the AMI with other known prognostic markers

**Figure 4:**
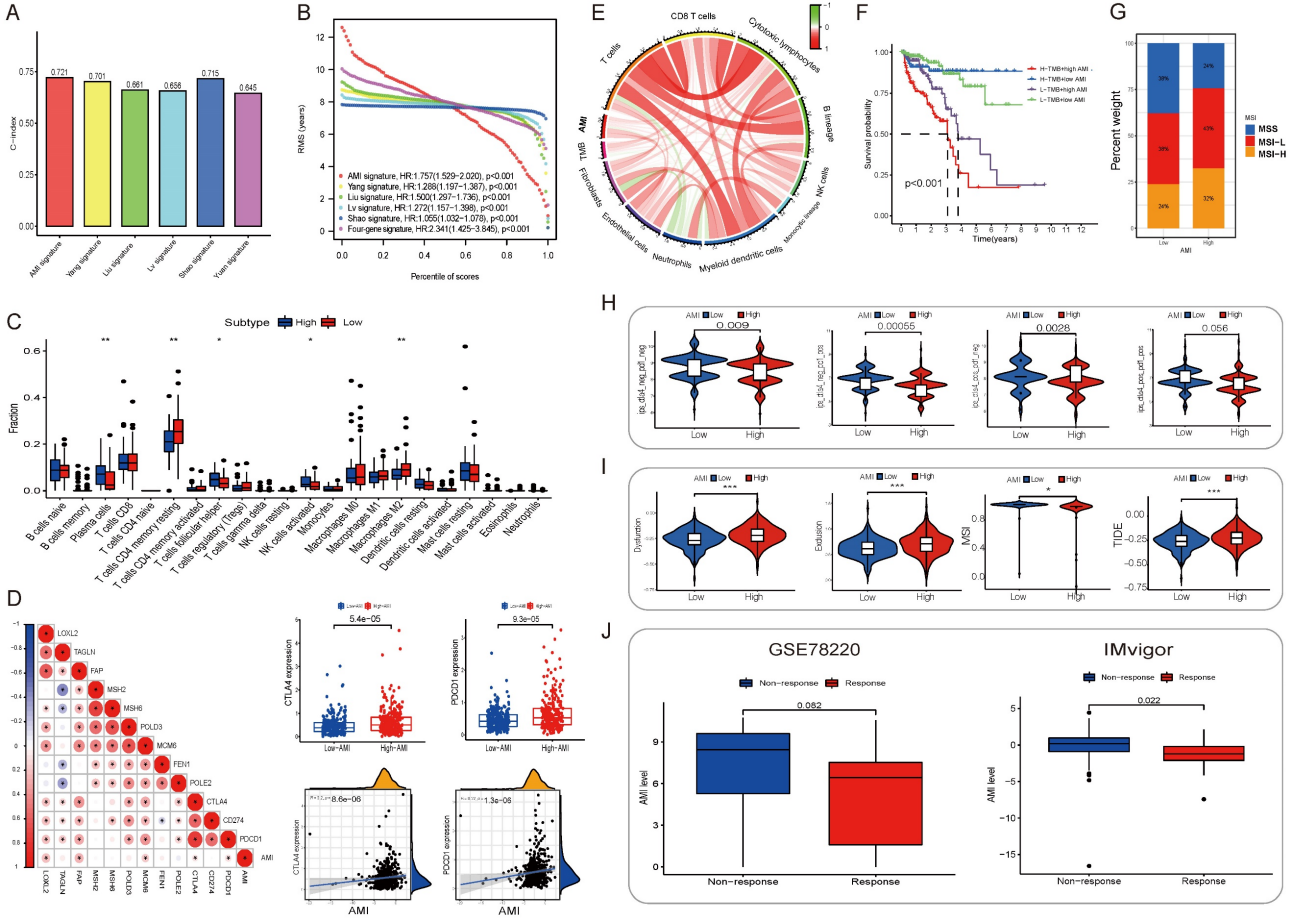
(A) The C-index of each model was calculated and compared. (B) The advantages and disadvantages of each model were compared by RMS curve. (C) The abundance of each TME infiltrating cell in high and low-AMI group. The upper and lower ends of the boxes represented interquartile range of values. The lines in the boxes represented median value, and black dots showed outliers. The asterisks represented the statistical p value (*P < 0.05; **P < 0.01; ***P < 0.001). (D) Correlations between AMI and the known immune checkpoints using Spearman analysis. The negative correlation was marked with blue and positive correlation with red. And we found that PD-1/L1 expression level in high AMI group were higher than low AMI group. (E) Correlations between AMI and TMB, the known immune cells using Spearman analysis. The negative correlation was marked with green and positive correlation with red. (F) Survival analyses for patients receiving anti-PD-L1 immunotherapy stratified by both AMI and TMB using Kaplan-Meier curves. H, high; L, Low; TMB, tumor mutation burden (P < 0.001, Log-rank test). (G)The proportion of molecular subtypes in high and low AM regulation patterns. (H) The relative distribution of IPS was also compared between AMI high and low groups in TCGA-PRAD cohort. (I) The relative distribution of TIDE was compared between AMI high versus low groups in TCGA-PRAD cohort. (J) Relationship between immunotherapy response and AMI level in two immunotherapy cohorts.

Several prognostic markers have been discovered for PCa using tumor tissues or in single-centre studies. “Signature lv” is considered as a promising tool to predict the BCR of PCa 26. “Signature Liu” is a ferroptosis-related gene signature developed to predict the prognosis of PCa^27^. “Signature Shao” is an independent index associated with OS of patients with PCa 28. “Signature Yang” comprising 28 hypoxia-associated genes serves as prognostic biomarkers for PCa^29^. “Signature Yuan” is a 4-gene signature developed for predicting the BCR of PCa based on clinical features 30. To compare the predictive accuracy of the AME signature with that of the abovementioned five signatures, ROC and c-index-based analyses of other biomarkers were performed in the TCGA cohort (**Fig. S9A-F; Fig. 4A-B**). The AME signature developed in this study was identified as a superior predictor and exhibited better stability and reliability in predicting the bRFS of patients with PCa.

### Exploration of potential mechanisms

To explore the underlying mechanisms of the AMI model, GSEA was used to analyse the potential biological processes and pathways. DEGs in the high- and low-AMI groups were primarily enriched in the p53 signalling pathway, IL-17 signalling pathway, apoptosis, extracellular matrix organisation, metabolism of important substances, androgen response and T cell activation (**Fig. S10B-C**). These results help deepen understanding of the cellular biological effects of the ageing microenvironment. Moreover, GSEA revealed that the high-AMI group was significantly enriched in cell adhesion molecules (CAMs), cytokine-cytokine receptor interaction and activation of immune response (**Fig. S10A**), whereas the low-AMI group was significantly enriched in propanoate metabolism, 'actin filament-based movement and actomyosin structure organisation (**Fig. S10A**). In addition, the expression of Ki67 was higher in the high-AMI group than in the low-AMI group (**Fig. S7C-F**).

### Immune infiltration and tumor microenvironment

To examine the relationship between the AME signature and tumor immunity, we used the ESTIMATE algorithm to analyze TCGA dataset. **Fig. S11A** in **Supplementary appendix** shows the specific correlation between each tumor-infiltrating cell type and eight regulators as assessed via Spearman's correlation analysis. We focused on two regulators, *COL1A1* and *BGLAP*, and observed their significantly positive correlation with most tumor-infiltrating immune cells. **Fig. 4C** shows the abundance of immune cell types in the low- and high-AMI groups in the training set. On performing an immune cell type-specific analysis, we found that patients with low AMI exhibited higher levels of resting memory CD4 T cells and M2 macrophages, whereas high-AMI patients exhibited higher levels of activated NK cells, follicular helper T cells and plasma cells (**Fig, 4C**). To further assess the characteristics of the AME signature, we analysed the correlation between several immune checkpoints and the AMI (**Fig. 4D**) and found that PDCD1 and CD274 showed a positive correlation with AMI (**Fig. 4D**, R_PDCD1-riskscore_ = 0.22, P < 0.001; R_CD274-riskscore_ = 0.2, P < 0.001). In addition, tumor mutation burden, which is closely associated with immunotherapeutic efficacy, was assessed. We found that patients with high AMI and mutation burden had a worse prognosis (**Fig. 4F; Fig. S11D-E**). Furthermore, we examined the specific relationship between the AMI and the abundance of eight tumor-infiltrating leukocytes and two stromal cell populations using MCP-counter analysis (**Fig. S11B**). We found that the AMI was positively correlated with CD8 T cells, cytotoxic lymphocytes, monocytes, endothelial cells and fibroblasts but negatively correlated with neutrophils. Moreover, tumor mutation burden was positively correlated with CD8 T cells (**Fig. S11B; Fig. 4E**), and the prognosis of patients with MSI-L and MSI-H status was poorer than that of patients with MSS, who also had a lower AMI value (**Fig. 4G; Fig. S11F-G**).

### Different AME landscape between AMI-high/low subgroup and relationship between eight hub AME regulators and AME

By analyzing the differences in the abundance of immune cell and fibroblast infiltration, immune function status and the levels of senescence related cytokines/inflammatory factors (including matrix metalloproteinases, *MMP1/3/10*; plasminogenactivator inhibitor 1/2, *PAI1/2*; chemokines/cytokines and growth factors, *CXCL1/2*, *IL6/8/10*, *IGFBP3/4/5/7*, *CSF2*; fibroblast related inflammatory factors, *HAPLN1*, *LOXL1*) between AMI-high and AMI-low groups, the results showed that the degree of immune cell infiltration was higher in the AMI-high group, the immune function was active, and a large amount of senescence related cellular inflammatory factors were secreted (**Fig. S12-13**). Further analysis of eight important aging regulatory factors in AMI showed that their expression levels were highly correlated with the score of immune matrix fibroblast, the degree of infiltration, the score of immune function status and the level of age-related cellular inflammatory factors. For example, *COL1A1* expression was significantly positively correlated with fibroblast infiltration abundance, and samples with high *KRTAP4-3* expression levels showed higher NK cell infiltration abundance. The expression level of *BGLAP* was negatively correlated with the infiltration abundance of neutrophils. In addition, the expression levels of *NR5A1* and *BGLAP* were positively correlated with the co-stimulatory state of T cells, while *PDCD4* and *ANXA3* showed the opposite trend. As for the regulation of inflammation and chemokines related to aging, *COL1A1* mainly regulates the secretion level of *IGFBP3/4/5/7*, *NR5A1/PITX3* mainly regulates the secretion level of *IL8/10/CSF2*, and the expression level of *ANXA3* is negatively regulated with these cytokines (**Fig. S14**).

### Nomogram for bRFS prediction

To improve the ability of the AMI model in predicting bRFS, we combined the AMI with conventional clinicopathological features and constructed a nomogram in the TCGA cohort (**Fig. S8A**). The ROC curve (AUC_5years_ = 0.846) showed good predictive accuracy of the nomogram (**Fig. S8D**). To further assess the predictive reliability and clinical applicability of the nomogram, we plotted calibration curves and performed DCA. The calibration curves illustrated good probability consistencies of the nomogram (**Fig. S8B**). DCA verified that the net benefit of prediction was higher in the nomogram model compared with the TNM staging system (**Fig. S8C**). In addition, the nomogram achieved the highest AUC value of all other clinicopathological parameters in predicting 5-year bRFS (**Fig. S8D**).

### Prediction for benefits of antiandrogenic therapy, chemotherapy and immunotherapy

**Figure 5:**
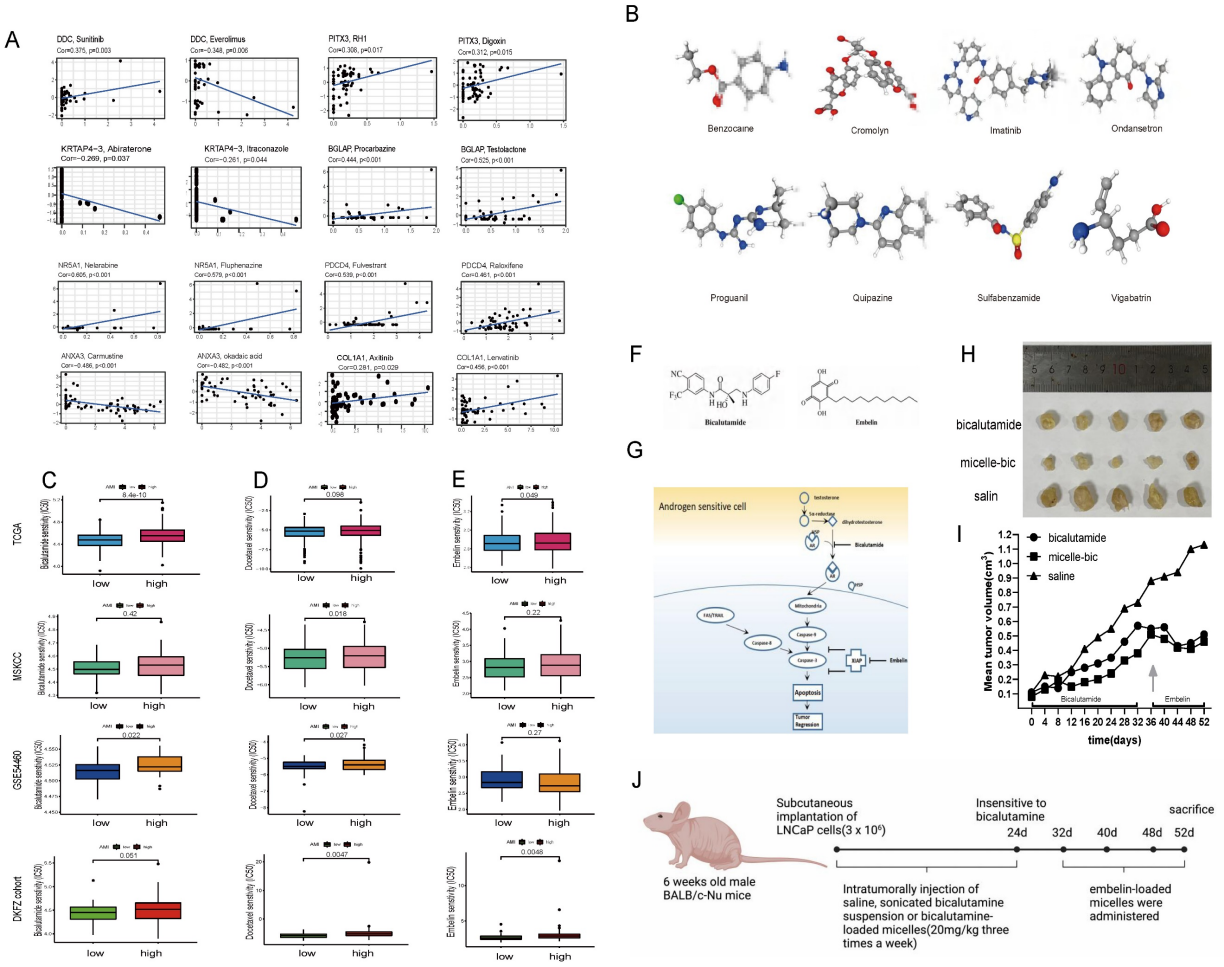
(A) CellMiner was used to screen for anticancer drugs and their targets based on the eight genes that were used to construct the model, and many anticancer drugs were significantly correlated with the expression of these genes. (B) The 3D structure tomographs of the eight candidate small-molecule drugs(benzocaine, cromolyn, imatinib, ondansetron, proguanil, quipazine, sulfabenzamide and vigabatrin) for PCa. (C) Sensitivity to bicalutamide in different risk groups. (D) Sensitivity to docetaxel in different risk groups. (E) Sensitivity to Embelin in different risk groups. (F-G) Chemical structures of bicalutamide and embelin (F) and schematic diagram showing the combined effect of bicalutamide and embelin on apoptosis in androgen-sensitive cells and on tumor regression (G). (H-I) Effect of bicalutamide and embelin-loaded micelles on growth of tumors derived from LNCaP prostate cancer cells in nude mice. Points are mean tumor size (n=5); bars, SE. (J) The flowchart of the in-vivo efficacy assessment of drug-loaded micelles in xenografts.

Additionally, alongside radical surgery and radiotherapy, endocrine therapy and chemotherapy are effective treatments for PCa. GSEA indicated significant enrichment of "androgen response" in the low-AMI group. We assessed the response to bicalutamide and found that the low-AMI groups in three cohorts responded better (P < 0.05), except for the MSKCC cohort (P = 0.42) (**Fig. 5C**).

Moreover, the high-AMI group had higher Gleason scores, indicating faster tumor growth, rapid cell proliferation, and poorer tissue differentiation. We investigated the response to chemotherapy and observed that the low-AMI group in four cohorts responded better to docetaxel (**Fig. 5D**). Additionally, apoptosis was enriched in cancer-related pathways, and the low-AMI group in TCGA and DKFZ cohorts showed a better response to embelin, an apoptosis inhibitor (**Fig. 5E**).

For immune therapy, TIDE scores were significantly lower and IPS was significantly higher in the low-AMI group (P < 0.001) (**Fig. 4H, 4I**). AMI levels differed significantly between non-responders and responders in two anti-PD-L1 immunotherapy cohorts (**Fig. 4J**), indicating the role of AME regulatory patterns in mediating the immune response.

Using CellMiner, we identified anticancer drugs correlated with the expression of the model's eight genes. Axitinib's activity correlated positively with *COL1A1* expression, while testolactone's activity correlated positively with *BGLAP* expression (**Fig. 5A**). Furthermore, using the CMap database, we identified eight small-molecule drugs with therapeutic potential for PCa patients based on upregulated and downregulated genes (**Table S3**). The 3D structure of these drugs was downloaded from PubChem (**Fig. 5B**).

### In vivo efficacy assessment of drug-loaded micelles in xenografts

During 20 days of bicalutamide treatment, tumor growth was effectively suppressed (**Fig. 5I**). Bicalutamide formulated into PEG-PLA polymeric micelles suppressed tumor growth more significantly. However, these tumors became resistant to bicalutamide after treatment for 20 days and began to grow again. Antiapoptotic proteins like XIAP is reported to be upregulated in hormone refractory prostate cancer, thus emblin, an effective XIAP inhibitor, was subsequently applied to these tumors. As exhibited in **Fig. 5F-J**, sequential treatment with embelin led to regression of bicalutamide resistant tumors.

### *COL1A1* and *BGLAP* knockdown inhibited PCa proliferation and migration and promoted apoptosis

To further investigate the relationship between the expression of AME regulators and PCa metastasis, we compared the distribution of 36 AME regulators in eight PCa cell lines with different pathological origins. Most regulators had higher distribution in metastasis-derived cell lines than in primary tumor-derived cell lines (**Fig. S15A**), indicating that modification of the ageing microenvironment may help to promote PCa metastasis. Particularly, two regulators, *COL1A1* and *BGLAP,* were significantly upregulated in metastasis-derived cell lines. Moreover, we determined that the two regulators acted as the pan-cancer fitness gene (**Fig. S15B**)^21^, and DepMap data also reminded that *COL1A1* and *BGLAP* were essential genes in almost all cancer types (**Fig. S16**). Therefore, we assessed whether these molecules could be therapeutic targets for metastatic PCa.

**Figure 6:**
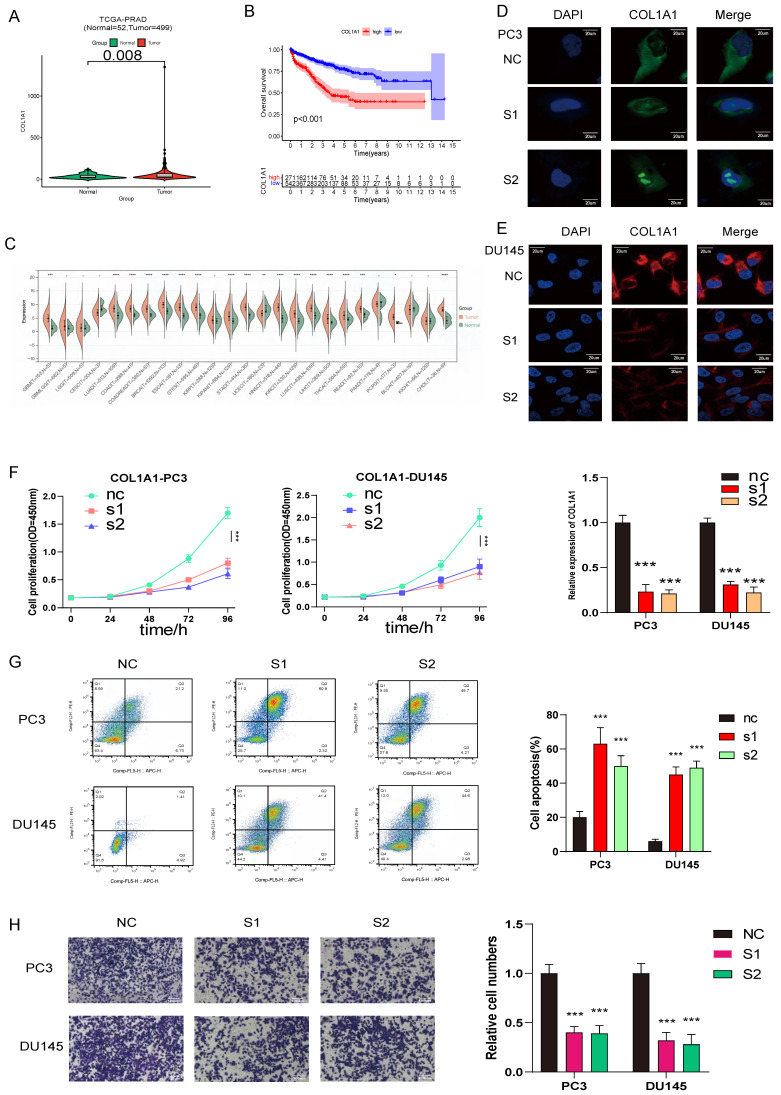
COL1A1 are upregulated in prostate cancer and promotes prostate cancer progression, COL1A1 positively related to PCa ISUP grading were verified in SYSU cohort by Immunohistochemistry (IHC) H score. (A) Compared with normal prostate tissue, COL1A1 is upregulated in prostate cancer. (B) The survival curves of COL1A1 expression was estimated by the Kaplan-Meier plotter. (P <0.001, Log-rank test). (C) The relative expression of COL1A1 between tumor and normal tisuue across pan-caners. (D-E) Immunofluorescence microscopy analysis shows the expression of COL1A1 in control and knockdown cells (PC3 and DU145, Scale bar, 20μm); Results of qPCR the knockdown (KD) efficiency of COL1A1. (F) The cell growth rate is evaluated in COL1A1-KD and control cells. (G) Apoptosis is determined in COL1A1-KD and control cells. (H) Transwell migration assays of the migration ability of prostate cancer cells (PC3 and DU145, magnification, Scale bar, 100μm) in the control or knockdown groups.

**Figure 7:**
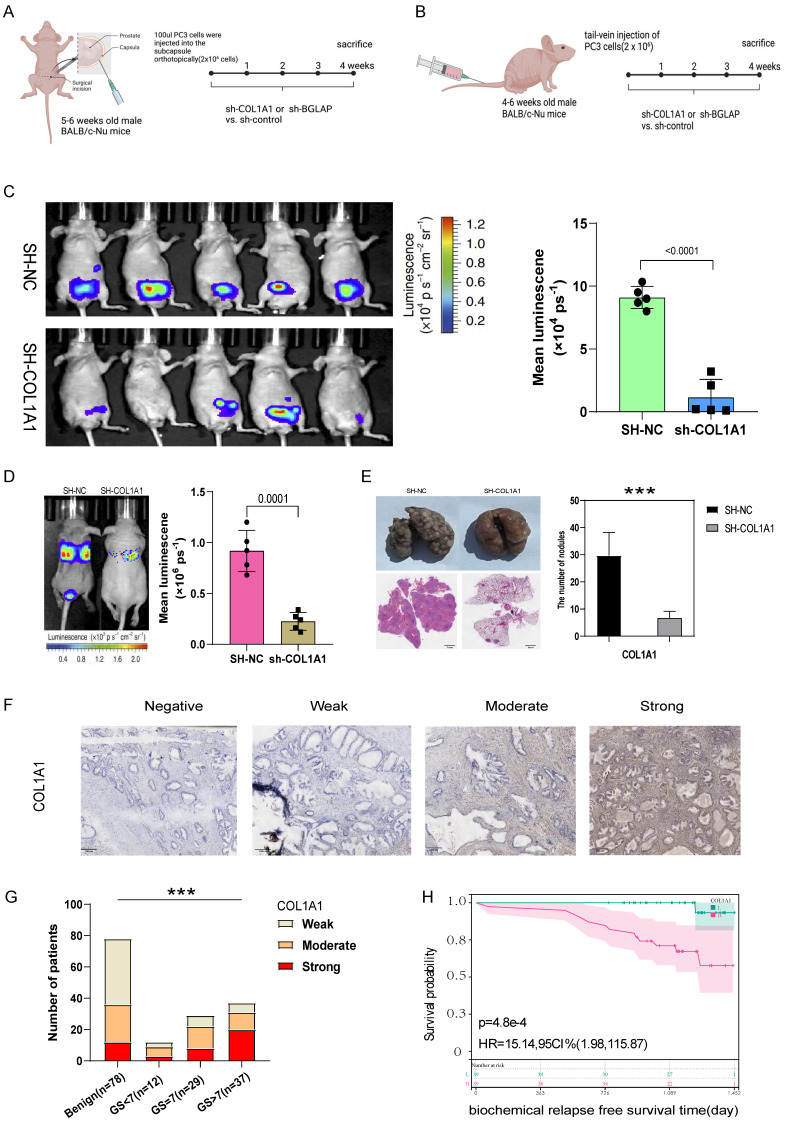
(A) The experimental flowchart of orthotopic-xenograft prostate-tumor mouse models. (B) The experimental flowchart of mouse models of pulmonary metastasis via tail vein. (C) Orthotopic-xenograft prostate-tumor mouse models implanted with COL1A1-KD PC3 cells. Representative bioluminescent images of orthotopic prostate tumors. Statistical calculation of the mean luminescence of the orthotopic xenograft tumors. (D) Bioluminescence of the lung metastatic nodules was detected by an in vivo bioluminescence imaging system. (E) Representative images of isolated lung tissues from the COL1A1_NC group and the COL1A1_SH group. Representative images of hematoxylin-eosin staining of lung slice from COL1A1_NC group and the COL1A1_SH group, Scale bar, 1mm. The number of metastatic nodules in the lungs from different groups. *, **, and *** represent P<0.05, P<0.01, and P<0.001, respectively. (F-G) The protein expression of COL1A1 in different Gleason score subtypes in PCa tissues by IHC, Scale bar, 100μm. (H) The survival curves of COL1A1 H score with were estimated by the Kaplan-Meier plotter. (P = 0.0073, Log-rank test). Comparison of bRFS between patients with a high H score and patients with a low H score was undertaken using the median value of the H score as the cutoff.

Then we hypothesised that *COL1A1* and *BGLAP* affected the malignant biological behaviour of PCa. We performed several experiments in vitro and in vivo with two metastasis-derived cell lines and observed that *COL1A1* and *BGLAP* knockdown significantly inhibited the proliferation, migration and metastasis of PCa cells and promoted apoptosis (**Fig. 6-7; Fig. S17-18**). In addition, we found that the protein expression of *COL1A1* and *BGLAP* was related to the ISUP grade of PCa in clinical samples (**Fig. 7D-F; Fig. S18D-F**). To further validate these findings, IHC analysis of these two genes was performed in 78 pairs of tumor tissues and the corresponding normal tissues after radical prostatectomy. Consistent with the expectation, the expression of *COL1A1* and *BGLAP* was higher in tumor tissues than in normal tissues, and high expression of *COL1A1* and *BGLAP* was associated with higher GS and BCR rate (P < 0.01) (**Fig. 7D-F; Fig. S18D-F; Table 2,3**). Besides, patients in the COL1A1_High&BGLAP_High group had a highest risk of biochemical recurrence (**Fig. S19A-B**). We also found in the TCGA dataset that the expression levels of COL1A1 and BGLAP were higher in the samples with positive surgical margins, and the differences were statistically significant (**Fig. S19C-D**). In the GSE54460 renal cell carcinoma dataset, the expression level of COL1A1 was higher in the positive surgical margin group, and the P-value was less than 0.05, but the expression level of BGLAP was not correlated with the positive surgical margin (**Fig. S19E-F**).

## Discussion

Recent studies have highlighted the synergistic effects of ageing-related changes in immune and stromal cell populations, leading to the progression of tumor cells. However, the comprehensive characteristics of the ageing microenvironment mediated by integrated AME regulators remain unclear. In this study, we identified three AME regulatory patterns (AME-A, AME-B, and AME-C) associated with distinct immune phenotypes (immune-desert, immune-inflamed, and immune-excluded). There has been a study revealing that TME plays an essential part in tumor progression and immunotherapeutic effect^31^. In addition, infiltrating levels of CD4+/CD8+ T cells, NK cells, M1 macrophages and inflammatory cytokines in tumors have been found related to the immune response degree^32^, ^33^.

We observed significantly elevated levels of tumor-infiltrating lymphocytes and PD-1/L1 in the AME-B pattern, suggesting their potential value for predicting immunotherapeutic benefits. A recent study has reported that the activation of EMT and TGF-β pathways impede lymphocyte cells from penetrating into the tumor parenchyma^34^. Specific molecular inhibitors targeting TGF-β were capable of reshaping TME (e.g. reprogramming of peritumoral stromal fibroblasts) and restoring anti-tumor immunity^35^, ^36^. Based on these evidences, we proposed that PCa patients with the AME-C pattern might benefit from the combination of ICB agents and TGF-β blockade treatment.

In addition to the establishment of the AMI quantification system, which accurately assesses patients' survival risk and guides personalized treatment strategies, we found that AMI was positively correlated with worse clinicopathological features and served as an independent risk factor for PCa patients. Remarkably, AMI was associated with MSI-H status, suggesting its potential as a preferable alternative to genomic aberrations. Considering the breakthrough in immune checkpoint inhibitor (ICI) therapy, we evaluated the association between AMI and predictors of immune response, including TMB, PD-L1, IPS, and TIDE scores^37^-^39^. We observed a significant correlation, indicating that AME regulators could influence the effectiveness of immunotherapy. Furthermore, the AMI demonstrated predictive value in distinguishing responders from non-responders in cohorts undergoing anti-PD-1/PD-L1 immunotherapy. To facilitate clinical decision-making, we developed an AMI-based nomogram for clinicians as a valuable reference tool. Significant attempts have been made in previous studies to develop models for predicting the prognosis of patients with PCa using RNA-sequencing data and clinical features^26^, ^28^, ^30^. However, few models have been practiced clinically. The nomogram we constructed showed superior discrimination and calibration, with an AUC value of 0.846 for predicting 5-year bRFS in the TCGA-PRAD cohort.

Most ARGs included in the signature are closely associated with the initiation, proliferation and metastasis of tumors. *COL1A1* is a promising biomarker and potential therapeutic target for hepatocellular carcinoma and facilitates the metastasis of breast and ovarian cancers^40^, ^41^. *BGLAP* is highly expressed in pancreatic cancer cells and promotes tumor growth and invasion^42^. However, few studies have described the role of *COL1A1* and *BGLAP* in PCa. We found that patients with higher stage had higher level of AMI. And more *COL1A1* and *BGLAP* are expressed in the tumor than normal prostate by analyzing the spatial transcriptomics data. Then experiments in vivo and in vitro were conducted to assess the influence of these two genes in PCa on proliferation, migration and apoptosis. The experimental results showed that *COL1A1* and *BGLAP* knockdown inhibited PCa proliferation and migration and promoted apoptosis.

In addition to *COL1A1* and *BGLAP*, the other six genes in the signature have been reported to be associated with cancers in both basic and clinical studies. A study of Hill et al. demonstrated that *DDC*-induced chronic inflammation could prompt rapid progression of intrahepatic cholangiocarcinoma^43^. *NR5A1* is an orphan nuclear receptor which is proved to be essential for sexual differentiation and development of multiple endocrine organs, as well as the proliferation of cancer cells^44^. Evidences have shown that *PDCD4* plays a critical role in the progression of several tumors^45^-^47^, and serves as a tumor suppressor in PCa to modulate tumor growth and castration resistance^48^. Furthermore, *PITX3* has been reported to be involved in mouse brain development^49^, and methylation of *PITX3* promoter is a prognostic biomarker for BCR-free survival in patients with PCa after radical prostatectomy^50^. *KRTAP4-3* is a keratin-associated protein involved in ageing and hair cycle in various tissues. However, specific mechanisms about how *KRTAP4-3* affects tumor progression have not been elucidated. *ANXA3* expression is related to the tumorigenesis and development of breast cancer^51^, and cancer-associated fibroblasts contribute to cisplatin resistance by regulating *ANXA3* in lung cancer cells^52^.

To better understand the underlying mechanisms of ARGs in mediating the differential prognosis of patients with PCa, we performed GSEA analyses and found that ARGs were involved in major cancer-related signaling pathways and immune related pathways. These results provide a foundation for further study on the mechanisms of ARGs in PCa. Besides, research suggests cellular senescence often triggers an immune response in TME^53^, and immune cell infiltration in TME promotes tumor growth^54^. However, the underlying mechanisms of ARGs in modulating immune cells are relatively unclear in PCa. In this study, the high-AMI group showed higher infiltration levels of activated NK cells, follicular helper T cells and plasma cells. However, low-risk patients exhibited higher infiltration levels of resting memory CD4 T cells and M2 macrophages. Future studies are required to explore the exact prognostic relevance of these cells.

To assess the response to chemotherapy and antiandrogen therapy, we evaluated the expression of Ki-67 and the response to androgen in relation to the high and low AMI groups. Our findings demonstrated that patients in the low-AMI group exhibited better responses to docetaxel and bicalutamide, which were consistent across the four independent PCa cohorts (TCGA, GSE54460, MSKCC, and DKFZ).

Additionally, we aimed to explore the therapeutic potential of a combined treatment involving an androgen receptor antagonist (bicalutamide) and a XIAP inhibitor (embelin) to suppress prostate cancer growth. To overcome the limitations associated with conventional solubilization drugs, we employed polymeric micelles for intratumoral injections in xenograft models. However, improvements are necessary for systemic administration, such as expanding the "cargo" space and selecting superior hydrophobic blocks in the copolymer to enhance drug solubilization. Overall, the AME cluster, AMI, and critical ARGs collectively contribute to the intricate interplay between the immune system, fibroblasts, immune function regulation, and the inflammatory milieu within the tumor microenvironment. Understanding these factors can provide insights into the immune response, tumor progression, and potential therapeutic strategies for cancer treatment.

Nevertheless, this study has a few limitations. Firstly, the predictive value of the AME signature in immunotherapy response should be validated in a prospective immunotherapy cohort. Secondly, considering the intratumoral heterogeneity and technical variations in transcriptome sequencing, the scRNA-seq data of samples should be increased in future clinical studies. Thirdly, the development of a standardized commercial gene detection kit based on the eight signature genes, facilitating automated AMI calculation, would be beneficial. Finally, the biological mechanisms underlying the signature genes, particularly *COL1A1* and *BGLAP*, remain unclear and require further investigation.

## Conclusions

In this study, we comprehensively evaluated the regulatory patterns of the tumor ageing environment in 813 PCa patients based on 36 AME regulators and found the AME distribution difference among these patterns. This integrated analysis revealed that dysregulation of the ageing microenvironment contributes to the regulation of tumor immunity. In addition, we constructed an 8-gene signature associated with the tumor ageing microenvironment, which can accurately predict the BCR of PCa. Patients with higher AMI were more likely to develop BCR, had higher clinicopathological stage and grade, and were less likely to respond to immunotherapy, chemotherapy and antiandrogen therapy. And our study demonstrated that the combination of XIAP inhibitor (embelin) and an androgen receptor antagonist (bicalutamide) can regress prostate cancer tumors. Besides, *COL1A1* and *BGLAP* can help to understand the underlying mechanisms of AME regulators in the progression of PCa.

## Supplementary Material

Supplementary methods, figures and tables.

## Figures and Tables

**Table 1:** Clinical features of all eligible 813 PCa patients from TCGA, GEO, MSKCC and DKFZ cohorts

Variables	TCGA cohort	GEO cohort	MSKCC cohort	DKFZ cohort
	(n = 495)	(n = 96)	(n = 140)	(n = 82)
Status: No BCR	409 (82.63)	51 (53.13)	104 (74.29)	64 (78.05)
BCR	86 (17.37)	45 (46.87)	36 (25.71)	18 (21.95)
Age<60	201 (40.61)	40 (41.67)	NA	NA
≥60	294 (59.39)	56 (58.33)	NA	NA
AJCC - T: T1	177 (35.75)	5 (5.21)	0	0
T2	203 (41.01)	66 (68.75)	86 (61.43)	56 (68.29)
T3	109 (22.02)	18 (18.75)	47 (33.57)	23 (28.05)
T4	3 (0.61)	7 (7.29)	7 (5.00)	3 (3.66)
TX	3 (0.61)	0	0	0
AJCC - N: N0	344 (69.49)	93 (96.88)	NA	NA
N1	78 (15.76)	3 (3.12)	NA	NA
Nx	73 (14.75)	0	NA	NA
AJCC -M: M0	453 (91.52)	93 (96.88)	NA	NA
Mx/M1	42 (8.48)	3 (3.12)	NA	NA
ISUP grade:1	45 (9.09)	11 (11.46)	41 (29.28)	11 (13.41)
2	145 (29.29)	52 (54.17)	53 (37.86)	50 (60.98)
3	101 (20.40)	21 (21.88)	23 (16.43)	11 (13.41)
4	56 (11.32)	7 (7.29)	11 (7.86)	1 (1.22)
5	148 (29.90)	5 (5.20)	12 (8.57)	9 (10.98)
Gleason score level: low	66 (13.33)	11 (11.46)	41(29.28)	11 (13.41)
mediun	183 (36.97)	73 (76.04)	76 (54.29)	61 (74.39)
high	246 (49.70)	12 (12.50)	23 (16.43)	10 (12.20)
Surgical margins				
positive	93 (18.79)	58 (60.42)	NA	NA
negative	313 (63.23)	33 (34.38)	NA	NA
unknown	89 (17.98)	5 (5.20)	NA	NA
Median follow-up, months (IQR)	14.10 (4.80-30.28)	53.57 (23.78-82.83)	45.56 (23.64-61.11)	35.61 (12.53-48.06)
Biochemical relapse free survival (95% CI)				
1 years	90.4 (88.9-91.9)	86.4 (82.9-89.9)	89.2 (86.6-91.8)	83.70 (79.6-87.8)
3 years	74.3 (71.3-77.3)	65.3 (60.4-70.2)	77.9 (74.2-81.6)	77.8 (73.0-82.6)
5 years	59.0 (54.5-63.5)	55.3 (50.1-60.5)	73.8 (69.6-78.0)	75.6 (70.5-80.7)

Note: Data are shown as n (%). AJCC, American Joint Committee on Cancer; GEO, Gene Expression Omnibus; PCa, prostate cancer; TCGA, The Cancer Genome Atlas; MSKCC and DKFZ cohort downloaded from http://www.cbioportal.org/datasets.

**Table 2:** Correlations between COL1A1 and BGLAP protein expression levels by H score and clinicopathological characteristics in PCa (SYSU cohort, n=78)

Characteristics	Classification	Cases	COL1A1 expression		P-value	BGLAP expression		P-value
			Low	High		Low	High	
Age (years)	< 65	27	65.69±5.97	67.56±7.36	0.076	65.82±6.06	67.44±7.31	0.154
	≥ 65	51						
PSA	<10	41	10.55±11.20	40.60±113.27	0.038*	10.50±11.80	40.65±113.19	0.018*
	10-20	20						
	>20	17						
BMI	<18.5	1	23.96±2.61	24.25±2.87	0.413	24.24±2.68	23.97±2.81	0.723
	18.5-24	39						
	24-28	31						
	≥28	7						
Biochemical Recurrence	NO	64	38	26	0.00048 ***	37	27	0.0063 **
	YES	14	1	13		2	12	
AJCC stage_T	T1	12	9	3	<0.0001 ****	12	0	<0.0001 ****
	T2	35	23	12		22	13	
	T3	24	6	18		4	20	
	T4	7	1	6		1	6	
WHO ISUP grading	1	13	13	0	<0.0001 ****	12	1	0.002 **
	2	15	7	8		6	9	
	3	13	7	6		8	5	
	4	23	8	15		10	13	
	5	14	4	10		3	11	
Intraoperative blood loss (ml)	<100	14	123.33±93.35	134.35±121.05	0.419	113.50±71.91	142.08±130.35	0.424
	100-300	26						
	≥300	4						
	NA	34						
Recovery time of urinary control (months)	<3	22	3.76±3.92	3.84±3.74	0.895	3.53±3.93	4.06±3.71	0.303
	3-6	27						
	≥6	14						
	NA	15						
Gleason score	6	12	12	0	<0.0001 ****	11	1	0.002 **
	7	29	16	13		15	14	
	8	23	6	17		9	14	
	9	14	5	9		4	10	

*P < 0.05 was considered to be statistically significant (chi-square test) AJCC, American Joint Committee on Cancer; SYSU cohort, 78 patients with PCa from Sun yat-sen University First affiliated Hospital

**Table 3:** Univariate and multivariate Cox regression analyses of different parameters on biochemical relapse-free survival in SYSU cohort (n=78)

Parameter	Univariate Analysis		Multivariate Analysis	
	HR (95%CI)	P Value	HR (95%CI)	P Value
Age (≥65 yr vs. <65 yr)	0.19 (0.06-0.6)	0.0017	0.19 (0.05-0.74)	0.02
T-Stage (III/IV vs. I/II)	0.56 (0.2-1.62)	0.003	2.33 (0.68-7.99)	0.18
Gleason Score (8/9 vs.6/7)	0.76 (0.43-1.35)	0.001	17.80 (1.35-234.11)	0.03
WHO ISUP (4/5 vs.1/2/3)	0.85 (0.57-1.28)	0.0016	0.05 (3.4e-3-0.71)	0.03
BGLAP expression level (High vs. Low)	6.22 (1.39-27.86)	0.0063	4.10 (0.76-22.00)	0.10
COL1A1 expression level (High vs. Low)	15.14 (1.98-115.87)	<0.001	17.24 (1.74-170.67)	0.01

HR=hazard radio. CI=confidence interval

## Abbreviations

PCa: prostate cancer
AME: ageing microenvironment
AMI: ageing microenvironment index
BCR: biochemical recurrence
ARGs: Ageing-related genes
TIME: tumor immune microenvironment
MSigDB: Molecular Signatures Database
TCGA: The Cancer Genome Atlas
PARD: Prostate adenocarcinoma
GEO: Gene Expression Omnibus database
ICGC: International Cancer Genome Consortium
DKFZ: Deutsches Krebsforschungszentrum
MSKCC: memorial sloan-kettering cancer center
CNVs: copy number variations
bRFS: biochemical relapse-free survival
FDR: false discovery rate
NMF: Non-negative Matrix Factorization
GSVA: gene set variation analysis
DEGs: differentially expressed genes
K-M: Kaplan-Meier
ROC: receiver operating characteristic
AUC: area under the ROC curve
UMAP: Uniform Manifold Approximation and Projection
PCA: principal component analysis
DCA: decision curve analysis
IPS: Immunophenoscore
TIDE: tumor Immune Dysfunction and Exclusion
CTLs: cytotoxic T lymphocytes
H-score: histologic score
PPI: protein-protein interaction
MDSC: Myeloid-derived suppressor cell
EMT: epithelial-mesenchymal transition
ISUP: International Society of Urological Pathology
PSA: prostate specific antigen
GS: Gleason Score
MSI: Microsatellite Instability
MSS: Microsatellite Stability
XIAP: X-linked inhibitor of apoptosis protein
CCLE: Cancer Cell Line Encyclopedia
ICB: immune checkpoint blockade
