# Supplementary material for: Therapeutic implications for localized prostate cancer by multiomics analyses of the ageing microenvironment landscape
Source: Int J Biol Sci. 2023 Jul 31;19(12):3951–69. doi: 10.7150/ijbs.85209 (PMC10411471; doi:10.7150/ijbs.85209)
Supplement: Supplementary file 1 — Supplementary methods, figures and tables. [file ijbsv19p3951s1.pdf]

Supplementary Appendix

Supplement to: Therapeutic implications for localized prostate cancer by multiomics analyses of the ageing microenvironment landscape

Table of Contents

Page

Supplementary Methods…………………………………………………………………..3

Cell culture……...……………..3

Cell viability and apoptosis assay…………………………..……........……………..3

RNA extraction and real-time reverse transcription polymerase chain reaction……..3

Immunofluorescence microscopy……...............…………………………………..……...4

Immunohistochemical analysis…………………………..……...............……………..4

Colony formation assay………………………........……………………………...……..5

Cell migration assay……….......................................…………………..…………………5

In vivo animal experiments…………........................………………..………………..6

Supplementary Figures…………………………………………………………………..8

Figure S1...……………………......…...8

Figure S2..……………………….........10

Figure S3..……………………….........11

Figure S4..……………………….........13

Figure S5..……………………….........15

Figure S6..……………………….........17

Figure S7..…………………......…...19

Figure S8..……………………......…..21

Figure S9..……………………….........22

Figure S10……………………………………23
Figure S11……………………………………25
Figure S12……………………………………27
Figure S13……………………………………29
Figure S14……………………………………31
Figure S15……………………………………33
Figure S16……………………………………35
Figure S17……………………………………37
Figure S18……………………………………39
Figure S19……………………………………41
Supplementary Tables……………………………………43
Table S1: Ageing related genes……………………………………43
Table S2: Univariate cox regression analyses of 18 bRFS-positive regulators and 18 bRFS-negative regulators……………………………………44
Table S3: CMap database was used to screen for top 30 small-molecule drugs……45
Table S4: Sequences of primer and siRNA used in this study……………………46

Supplementary methods:

Cell culture

PCa cell lines including PC3, DU145 and LNCaP were obtained from the American Type Culture Collection (Manassas, VA, USA) and cultured in RPMI-1640 medium containing 2-mM L-glutamine, 10% foetal bovine serum and 1% penicillin/streptomycin in a humidified incubator with 5% CO_2 at 37℃. Lentiviral vectors carrying *COL1A1* shRNA, *BGLAP* shRNA and the control shRNA were synthesised by and obtained from GenePharma (Suzhou, China).

Cell viability and apoptosis assay

The cell proliferation rate was assessed by Cell Counting Kit-8 (CCK-8; Dojindo, Japan) every 24h through absorbance measurement at 450 nm using a plate reader (VARIOSKAN LUX, Thermo Fisher Scientific, USA). Flow cytometry was applied to evaluate apoptosis. After being washed twice with cold PBS, cells were resuspended in the Annexin V binding buffer at a concentration of 10^6 cells/mL and cultivated with AlexaFluor 647 Annexin V (Biolegend, USA) at 4 °C for 15 min away from light. Subsequently, cells were added with PI (Sigma, USA) and then immediately analysed via flow cytometry (FACSCanto II, BD, USA).

RNA extraction and real-time reverse transcription polymerase chain reaction

Total RNA was isolated from cells with the Trizol reagent (Invitrogen, Waltham, MA, USA), of which approximate 500 ng RNA was applied for reverse transcription using the PrimeScript RT Master Mix (Takara Biotechnology [Dalian] Co., Ltd., Japan). Real-time reverse transcription polymerase chain reaction (qRT-PCR) was conducted using Premix Ex TaqTM II (Takara Biotechnology [Dalian] Co., Ltd.) on the Roche Light Cycler 480 Real-Time PCR system, using GAPDH for internal reference. The sequences of primers used are listed in **Supplementary Table S4**.

Immunofluorescence microscopy

To detect the protein expression of two AME regulators (*COL1A1* and *BGLAP*) before and after knockdown, PC3 and DU145 cells were fixed, permeabilised and prehybridised. Subsequently, the cells were incubated in the blocking buffer (PBST with 5% bovine serum albumin) at room temperature (RT) for 30 min, primary antibodies (1:200 dilution) at RT for 1 h and then secondary antibodies conjugated with Alexa Fluor 488- or 594- (1:200 dilution, Cell Signaling Technology) at RT for 1 h away from light, followed by incubation with DAPI (Vector Laboratories) for 10 min. The immunofluorescence images were generated using the confocal microscope. Antibody information is listed in **Supplementary Table S4**.

Immunohistochemical analysis

PCa tumor tissues and adjacent normal prostate tissues from 78 patients performed radical prostatectomy at the First Affiliated Hospital, Sun Yat-sen University, were examined via immunohistochemical (IHC) analysis. Tissue sections were incubated with

anti-*COL1A1* and anti-*BGLAP* (1:100 dilution), and the staining intensity was estimated using a histologic score (H-score) system via digital pathology image analysis. The H-score of each sample ranged from 0 (no staining) to 300 (maximum immunoreactivity) and was calculated based on immunostaining intensity and the corresponding percentage. In particular, the staining intensity was assessed by the ranking from 0 to 3, with 0, 1, 2, 3 representing negative staining, weak staining, moderate staining and strong staining respectively. Thereafter, the H-score was calculated according to the following formula: H-score = 3 × (% at 3) + 2 × (% at 2) + 1 × (% at 1). The experiments were reviewed and approved by the Ethics Committee of the First Affiliated Hospital, Sun Yat-sen University (GZJZ-SB2020-027). Written informed consent were provided by all patients participating in this study.

Colony formation assay

1×10^3 PCa cells in the logarithmic growth phase were seeded into 6-well plates and cultured for 7-10 days. When the number of cells in most single clones was over 50, cells were washed with PBS (dissolved in methanol), fixed with 4% paraformaldehyde for about 30 mins and stained by 0.1% crystal violet, then the number of colonies was quantified.

Cell migration assay

Cell migration assay was conducted using a transwell system with a 24-well inserted plate (5.0-μm pore size) following the manufacturer's instructions. The suspension of PC3 and DU-145 cell lines (1×10^5 cells/well) in 200 μL of FBS-free RMPI-1640 was

added to the upper chamber. Simultaneously, 800 μL of RMPI-1640 (with 20% FBS) was added to the lower chamber. After being incubated for 24 h, cells attached to the lower chamber were fixed and then stained with 0.1% crystal violet for about 20 min.

In vivo animal experiments

All animal experiments were approved by the Institutional Ethics Committee of the First Affiliated Hospital, Sun Yat-sen University. To assess the efficacy of bicalutamide-loaded micelles in vivo, three million LNCaP cells suspended in 1:1 media and matrigel were injected subcutaneously to 6-week old male BALB/C nude mice to induce xenograft flank tumors. When tumors grew to about 150 mm^3, mice were classified into three groups of five mice randomly to reduce differences in tumor size and weight. Mice in the three groups were injected intratumorally with saline, sonicated bicalutamide suspension and bicalutamide-loaded micelles (20 mg/kg) respectively three times a week. The sizes of tumors were measured by a caliper before each injection, and tumor volumes were calculated with the formula: ($width^2 \times length$)/2.

To examine the therapeutic potential of targeting COL1A1 and BGLAP in patients with PCa, an in vivo lung colonisation assay was performed. First, stable cell lines (PC3) with COL1A1 or BGLAP knockdown were established. Subsequently, 20 mice were split into four groups (COL1A1-NC, COL1A1-SH, BGLAP-NC and BGLAP-SH; n = 5), and 2×10^6 PC3 cells were transfected with the corresponding plasmid via tail-vein injection. After approximately 6–8 weeks, the mice were sacrificed using CO2 as an anaesthetic, and their lungs were removed. The lung tissues were soaked in picric acid and embedded in paraffin for H&E staining. Representative nodule images were captured after H&E staining. We also establish the orthotopic-xenograft prostate-tumor mouse models to

further survey the effect of promoting tumor growth.

Supplementary Figures:

FigureS1

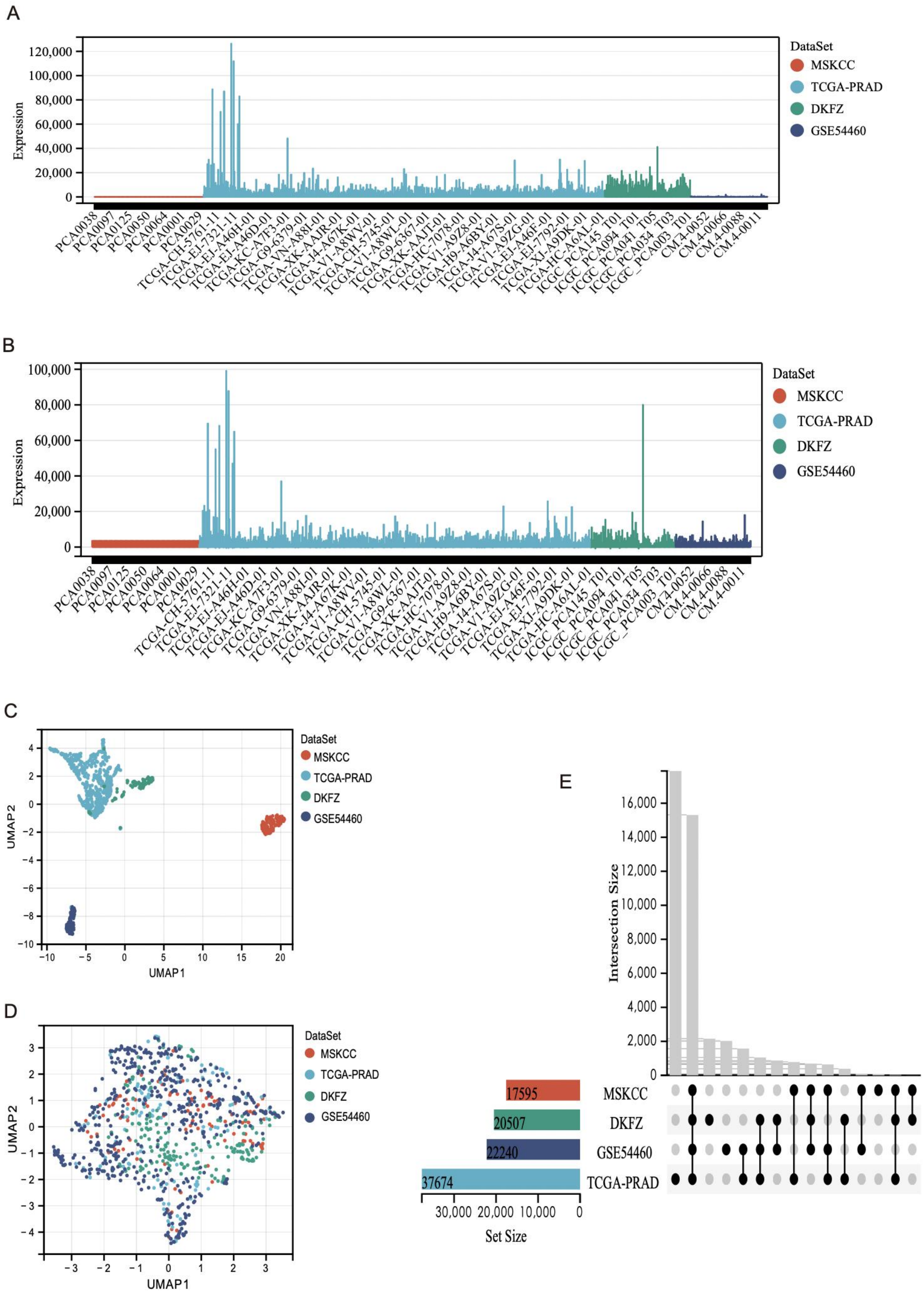

Fig. S1 Merge multiple datasets and remove batch effect. (A) From the boxplot, we can observe that the sample distribution of each data set differs greatly before the removal of batch effect, indicating the existence of batch effect. (B) After the removal of batch effect, the data distribution of each data set tends to be consistent, and the median is on the same line. (C) From the UMAP, we can observe that before the removal of batch effect, the samples of each data set were clustered together, indicating the existence of batch effect. (D) After the removal of batch effect, the samples of each data set were clustered and intertwined, indicating the removal of batch effect. (E) Venn diagrams by UpSetR: mRNA distributions in four data sets.

FigureS2

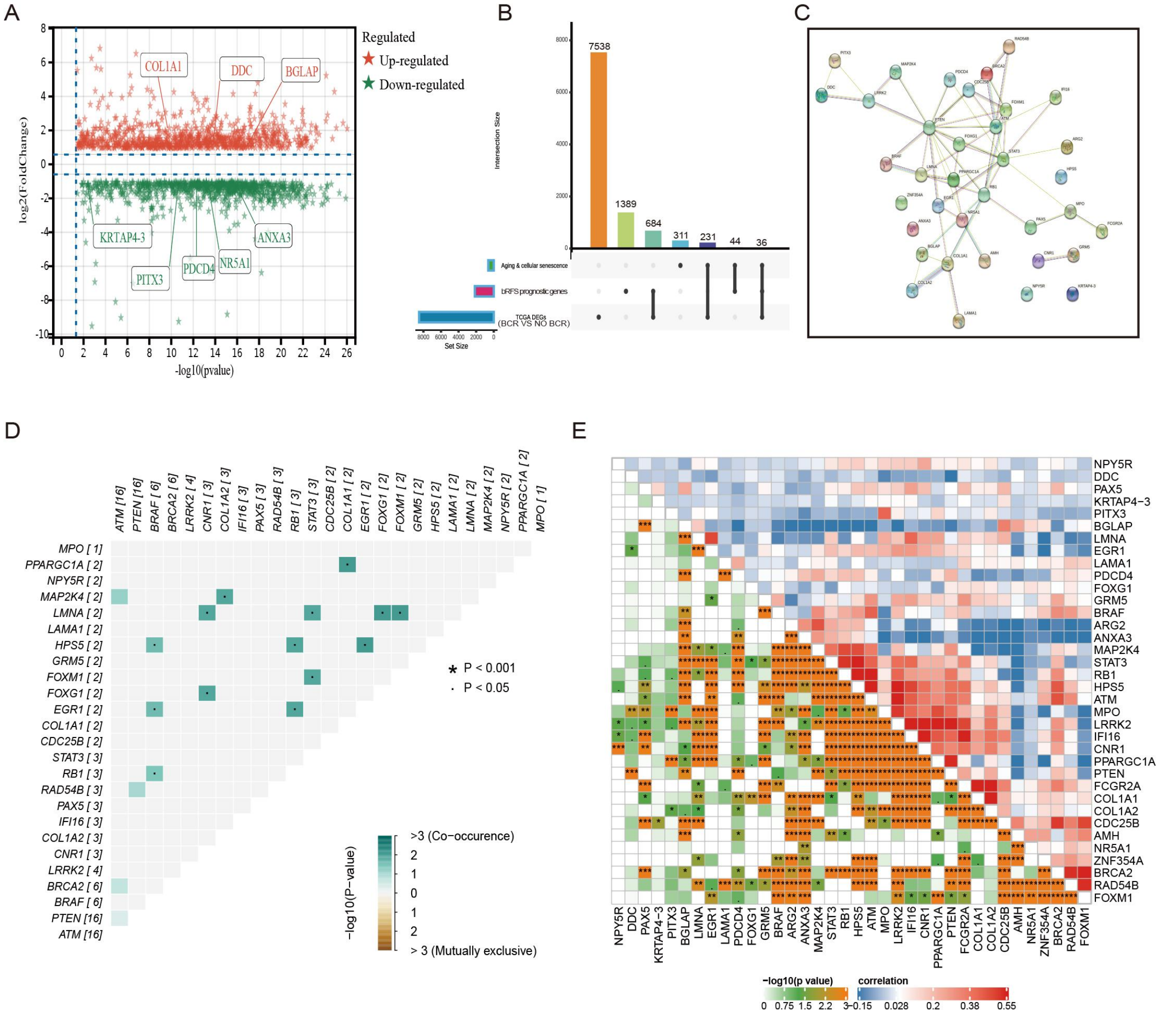

Fig. S2 (A) Volcano plot of differential expression of ageing-related genes between prostate cancer (PCa) tissues and normal tissues.(B) To obtain the important AME regulators by UpSetR (C) The construction of protein–protein interaction (PPI) network by STRING. (D) Co-occurrence of genetic alterations of the AME regulators in prostate cancer. (E) The correlation among AME regulators in prostate cancer. *P < 0.05, **P < 0.01, ***P < 0.001, ****P < 0.0001. ns, not significant.

Figure S3

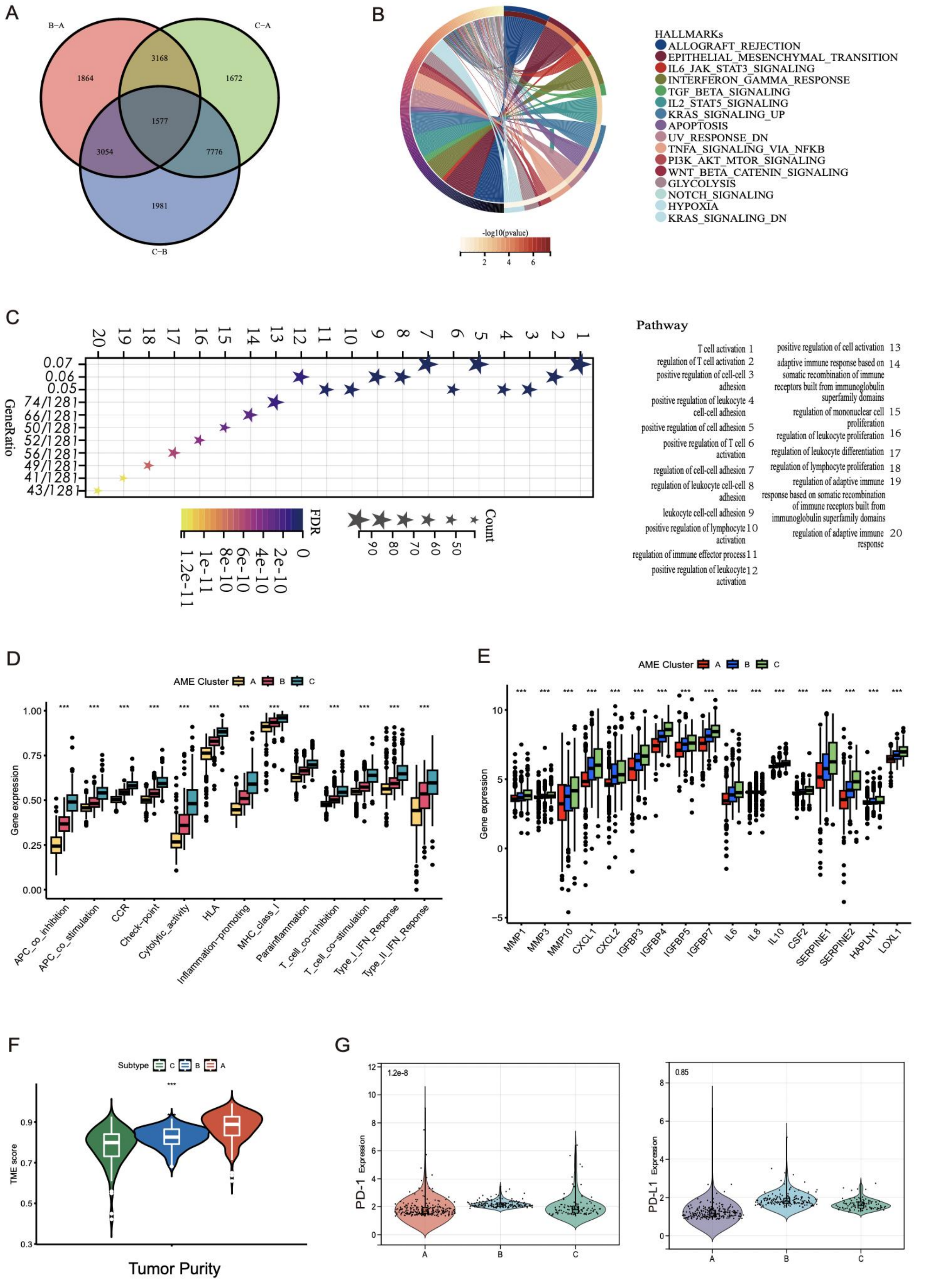

Fig. S3 (A) 1577 AME-related differentially expressed genes (DEGs) between three

AME-clusters were shown in the Venn diagram. (B-C) Functional annotation for AME-related genes using GO and GSEA enrichment analysis. The color depth of the barplots represented the number of genes enriched. (D) Boxplot showing differences in the immune function between three distinct AME clusters. (E) Boxplot showing differences in the expression levels of senescence related cytokines/inflammatory factors between three distinct AME clusters. (F) Estimate score of tumor purity between High AMI and Low AMI group. (G) Comparison of PD-1/L1 expression level across three AM regulation patterns.

Figure S4

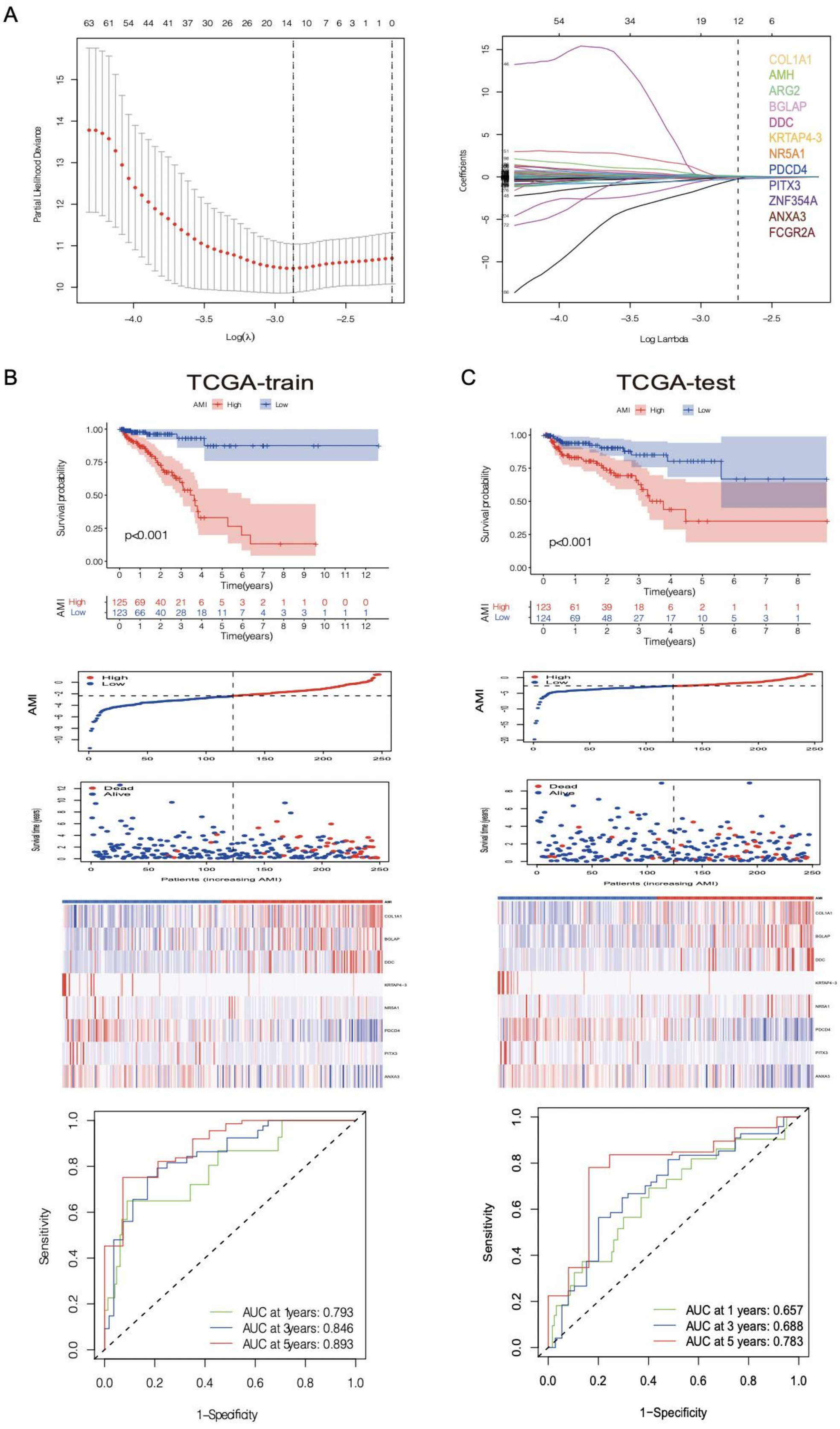
A
B
C
TCGA-train
TCGA-test
Partial Likelihood Deviance
Log(λ)
Coefficients
Log Lambda
COL1A1
AMH
ARG2
BGLAP
DDC
KRTAP4-3
NR5A1
PDCD4
PITX3
ZNF354A
ANXA3
FCGR2A
Survival probability
Time(years)
p<0.001
AMI
High
Low
Dead
Alive
Survival time (years)
Patients (increasing AMI)
Sensitivity
1−Specificity
AUC at 1 years: 0.793
AUC at 3 years: 0.846
AUC at 5 years: 0.893
AUC at 1 years: 0.657
AUC at 3 years: 0.688
AUC at 5 years: 0.783

Fig. S4 Construction of the the ageing microenvironment index(AMI). (A) Illustration for LASSO coefficient profiles of 36 AME regulators; Cross-validation was conducted for tuning parameter selection in the LASSO regression model. LASSO, least absolute shrinkage and selection operator; (B-C) The distribution of AMI, biochemical recurrence status along with bRFS times of PCa patients and heatmaps of 8 key prognostic AME regulators; Kaplan–Meier survival curves of bRFS and ROC analysis of the AME signature indicated that the signature has good bRFS predictive. (B) Training group, (C) testing group.

Figure S5

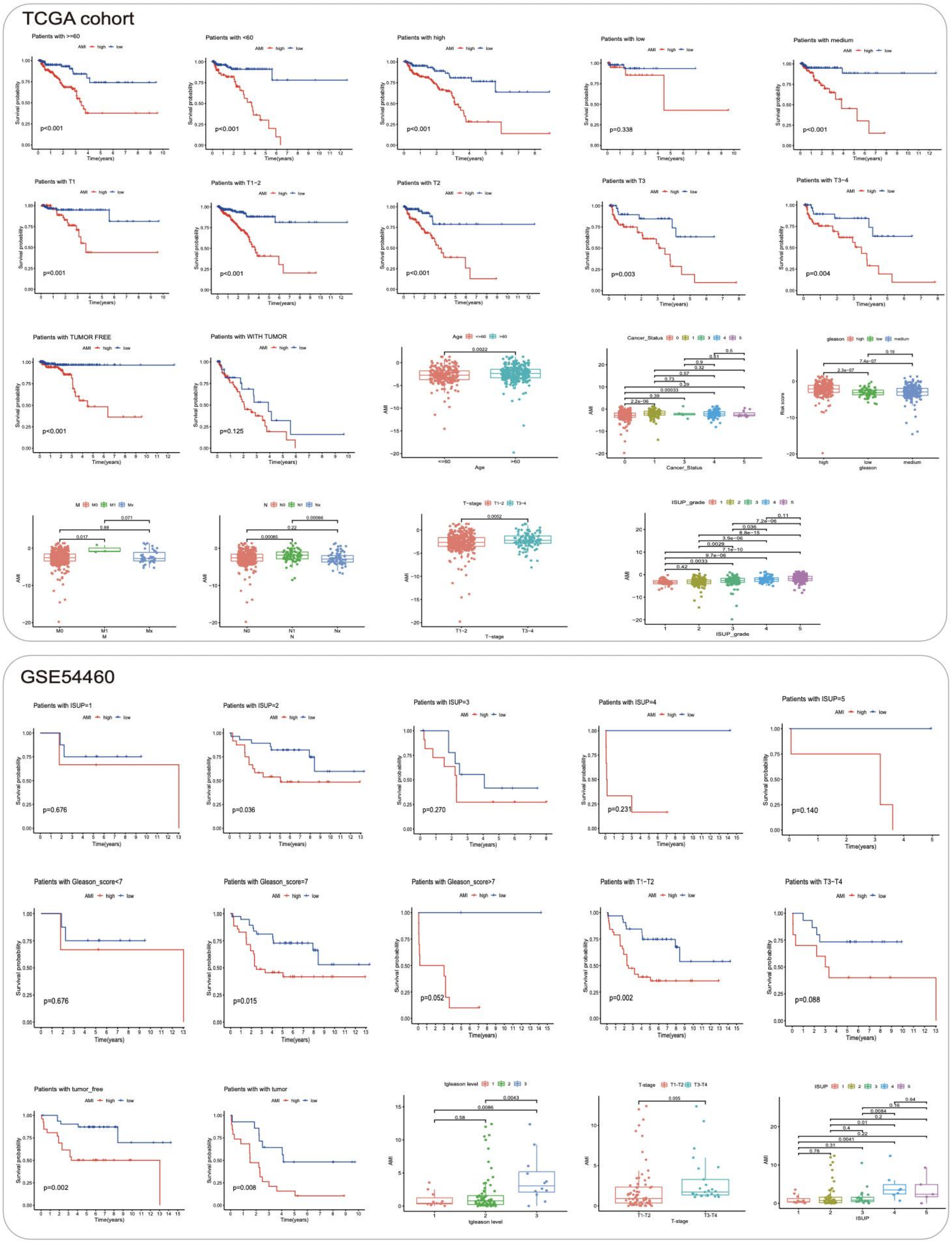
TCGA cohort
Patients with >=60
Patients with <60
Patients with high
Patients with low
Patients with medium
Patients with T1
Patients with T1−2
Patients with T2
Patients with T3
Patients with T3−4
Patients with TUMOR FREE
Patients with WITH TUMOR
GSE54460
Patients with ISUP=1
Patients with ISUP=2
Patients with ISUP=3
Patients with ISUP=4
Patients with ISUP=5
Patients with Gleason_score<7
Patients with Gleason_score=7
Patients with Gleason_score>7
Patients with T1~T2
Patients with T3~T4
Patients with tumor_free
Patients with tumor

Fig. S5 Assessment the prognostic roles(bRFS) of the AME signature via stratification of patients based on specific demographic and clinical features in the TCGA-PRAD, GSE54460 cohort. Age $\geq$ 60 years vs. Age < 60 years; Gleason score level:high;medium;low. Stage:T1-T4. surgical margins： positive; negative. ISUP grading:1-5.

Figure S6

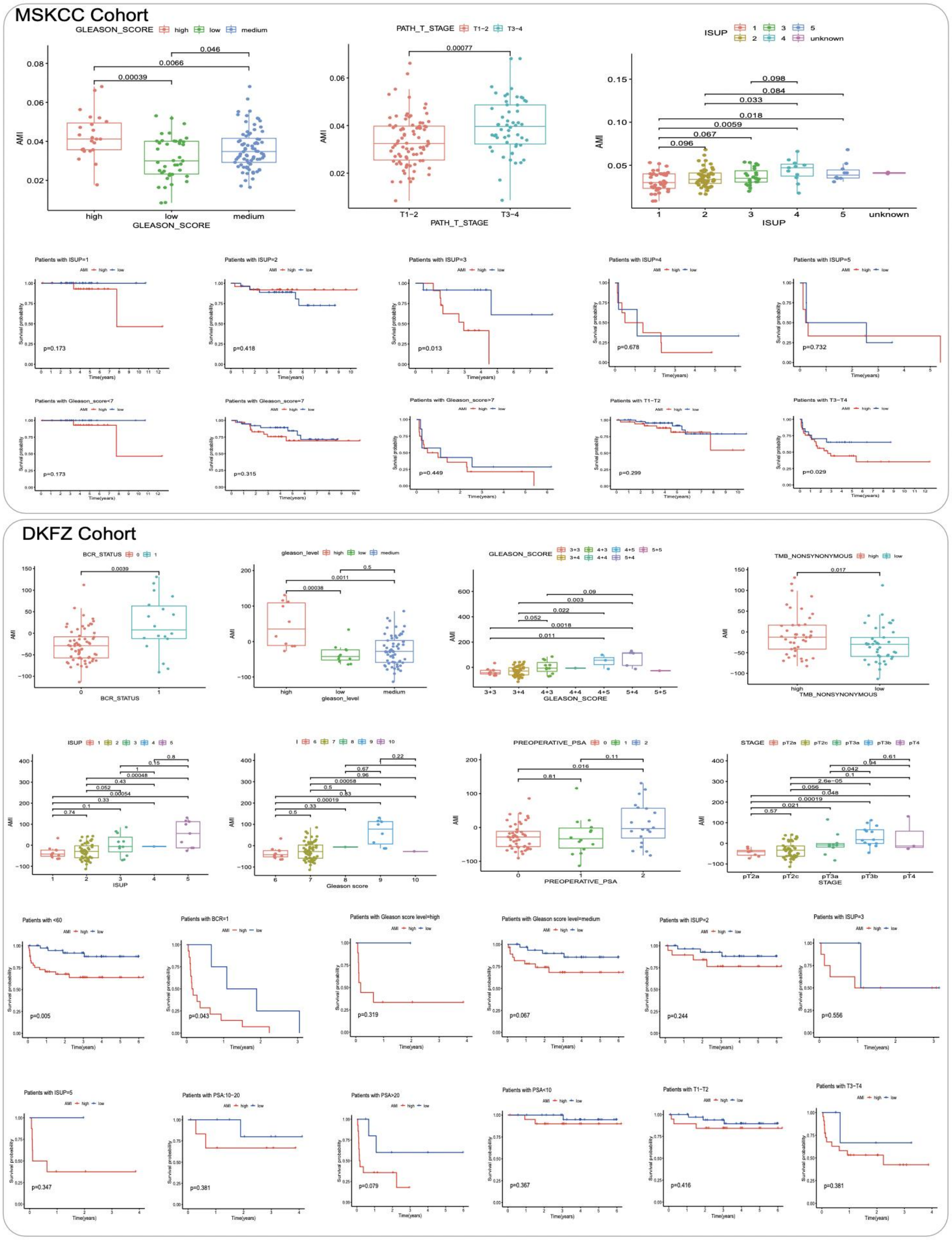

Fig. S6 Assessment the prognostic roles(bRFS) of the AME signature via stratification of patients based on specific demographic and clinical features in the MSKCC cohort and

DKFZ cohort. Age ≥ 60 years vs. Age < 60 years; Gleason score level:high;medium;low. Stage:T1-T4. surgical margins： positive; negative. ISUP grading:1-5.

Figure S7

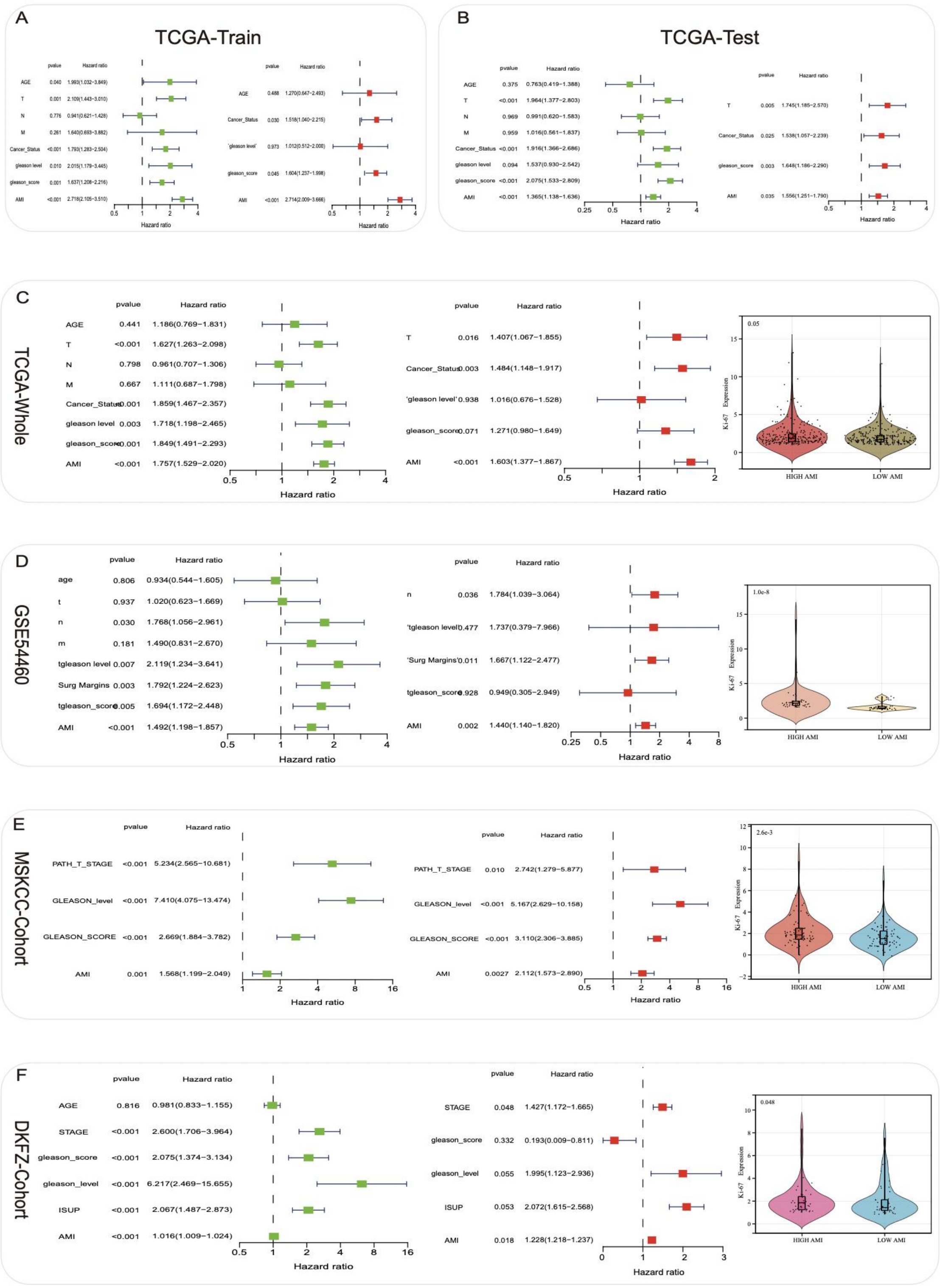

Fig. S7 Univariate and Multivariate Cox regression analysis and Nomogram for bRFS

prediction. (A-F) Univariate and Multivariate Cox regression analysis of the AME signature with bRFS in the four sets. The Ki67 expression level was higher in high-AMI patients.

Figure S8

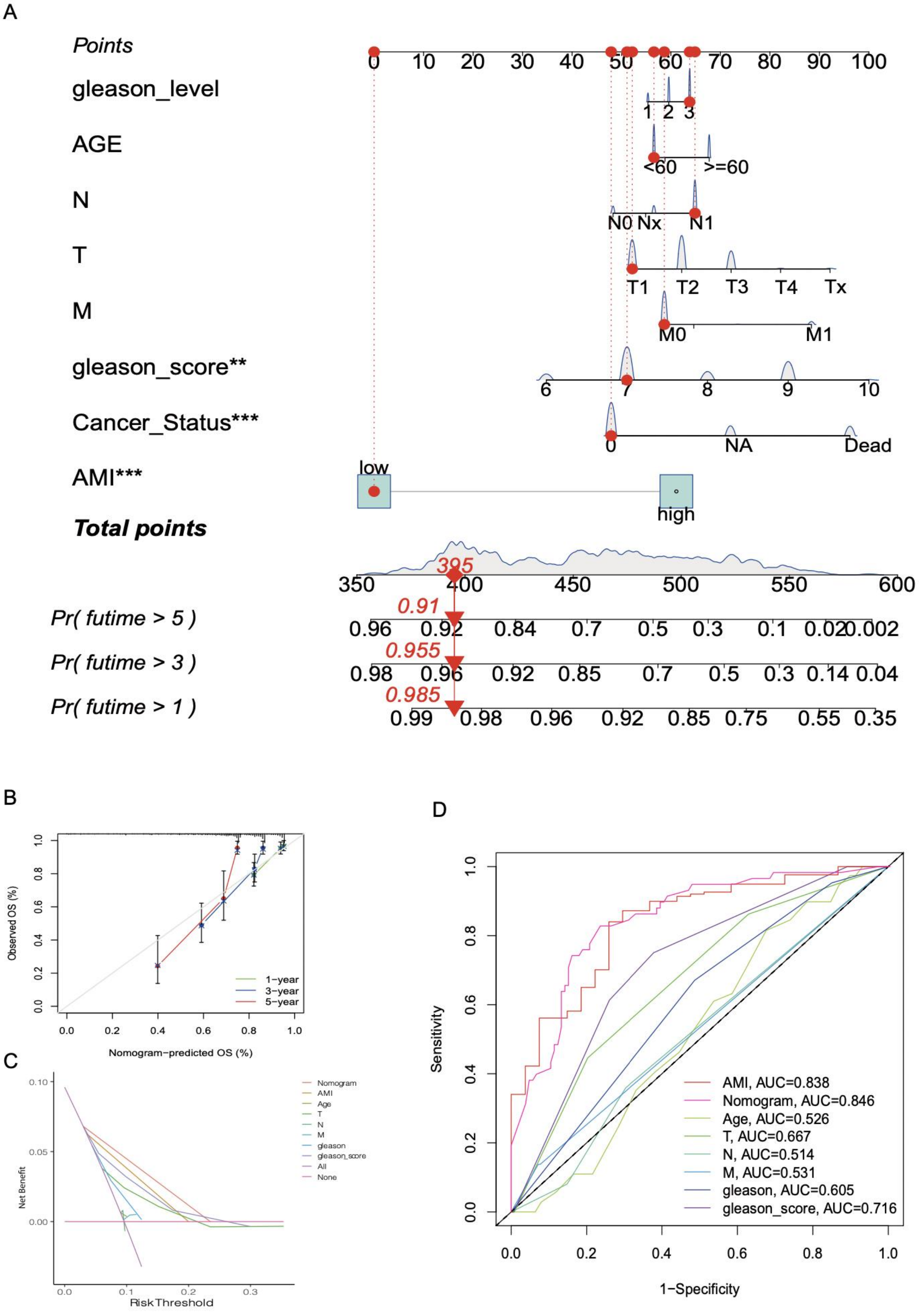

Fig. S8 (A) A prognostic nomogram including signature AMI and other clinical factors.

(B) The calibration curves of the 1-, 3-, and 5-year bRFS. (C) Decision curve analysis (DCA) was performed to assess the clinical utility of the AME signature. (D) ROC curve used to evaluate the 1-, 3-, and 5-year bRFS predictive efficiency.

FigureS9

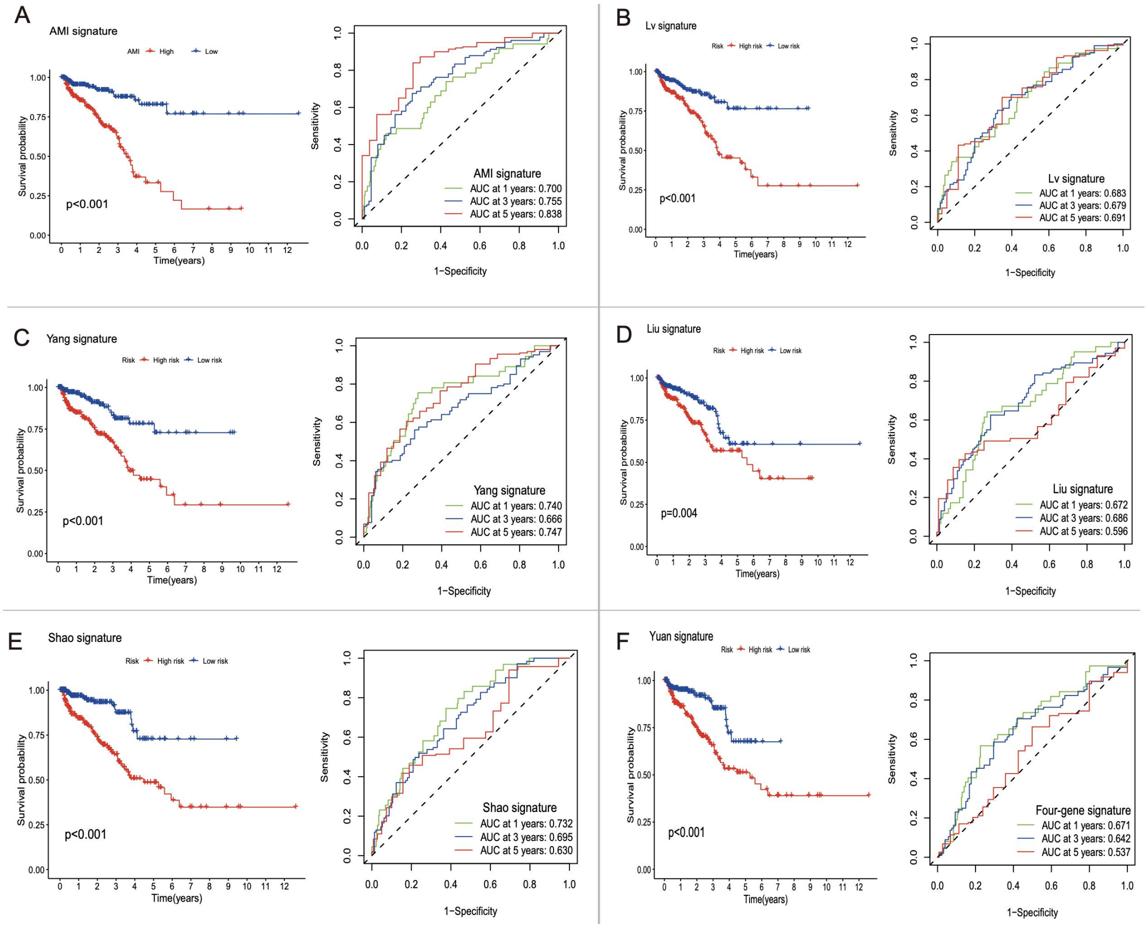

Fig. S9 Comparison of the AME signature with other known prognostic signatures. Kaplan–Meier survival curves of bRFS and ROC analyses of different prognostic signatures.

FigureS10

A

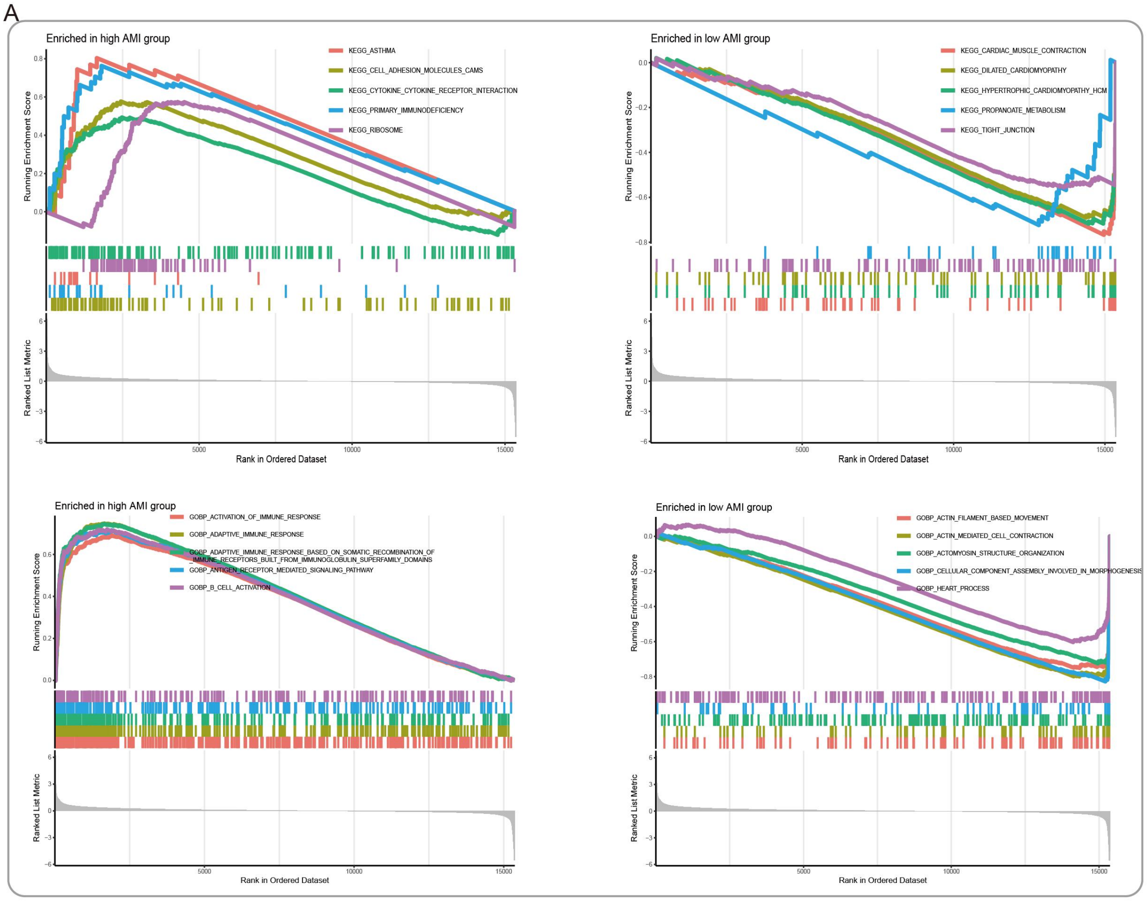

B

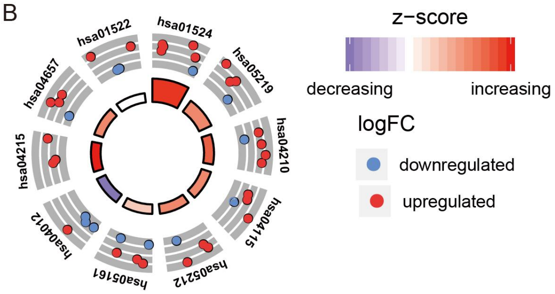

ID	Description
hsa01524	Platinum drug resistance
hsa05219	Bladder cancer
hsa04210	Apoptosis
hsa04115	p53 signaling pathway
hsa05212	Pancreatic cancer
hsa05161	Androgen response
hsa04012	ErbB signaling pathway
hsa04215	Apoptosis − multiple species
hsa04657	IL−17 signaling pathway
hsa01522	Endocrine resistance

C

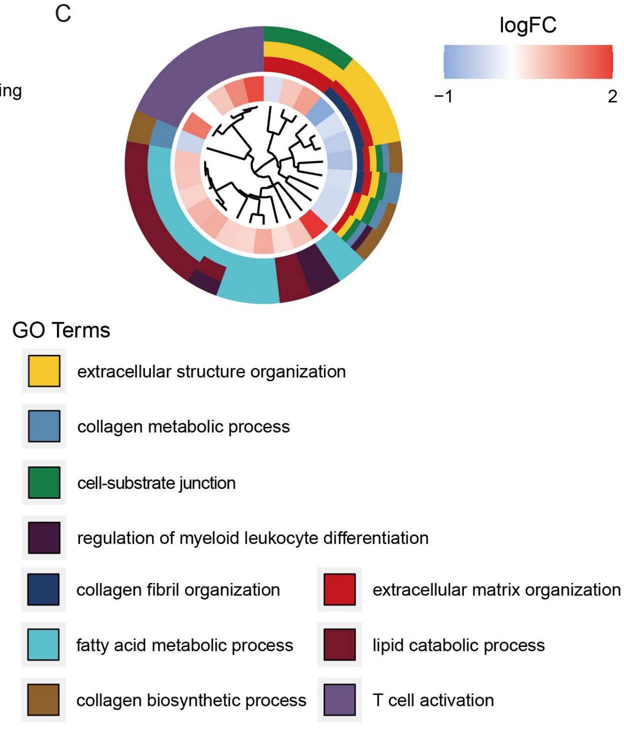

Fig. S10 The exploration of potential mechanism about the AME signature in TCGA cohort. (A) Gene set enrichment analysis (GSEA) results of the high and low-AMI groups. (B-C) Functional enrichment analysis by GO and KEGG based on differently expressed genes between high AMI versus low AMI.

FigureS11

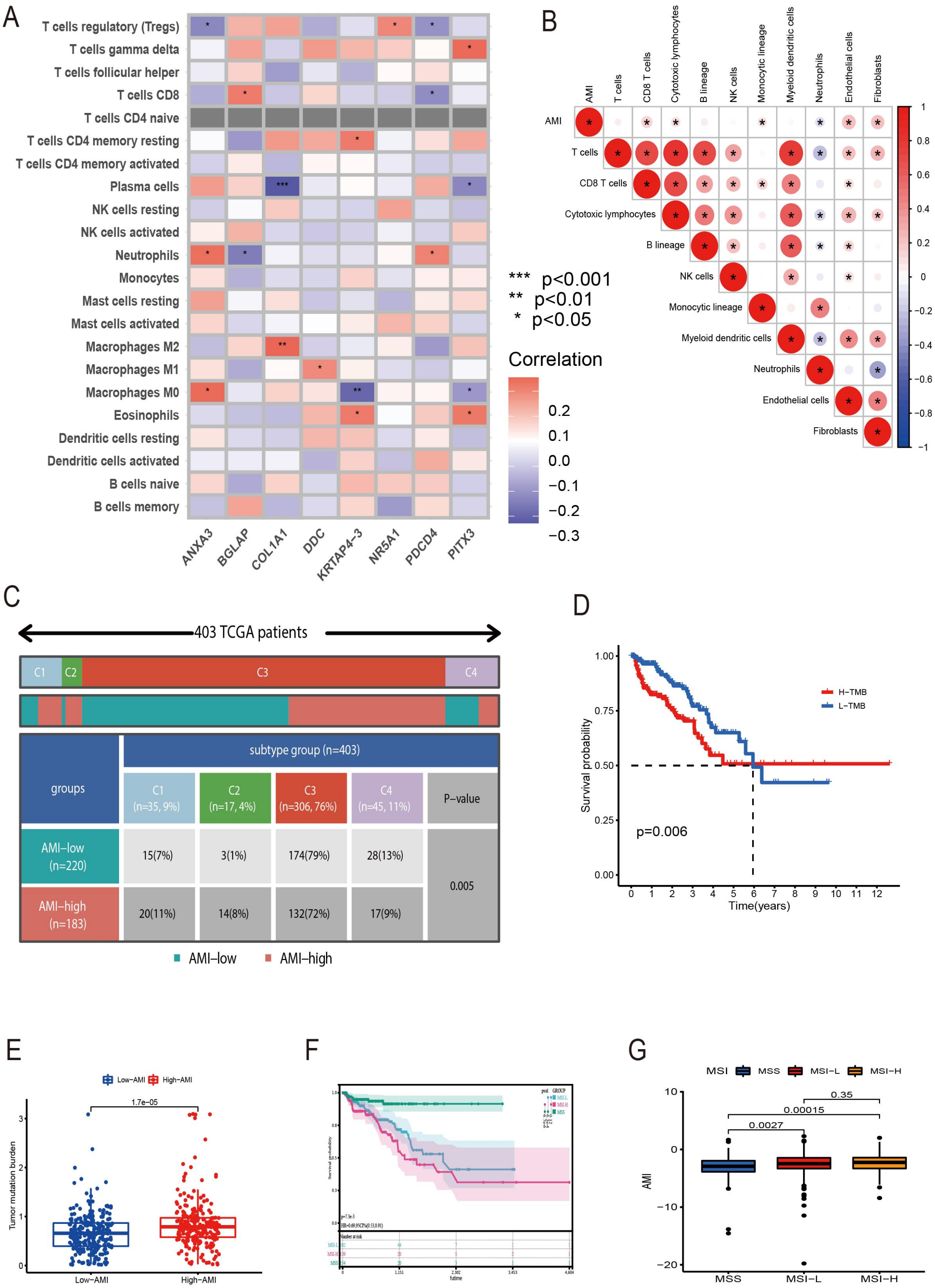
A
T cells regulatory (Tregs)
T cells gamma delta
T cells follicular helper
T cells CD8
T cells CD4 naive
T cells CD4 memory resting
T cells CD4 memory activated
Plasma cells
NK cells resting
NK cells activated
Neutrophils
Monocytes
Mast cells resting
Mast cells activated
Macrophages M2
Macrophages M1
Macrophages M0
Eosinophils
Dendritic cells resting
Dendritic cells activated
B cells naive
B cells memory
ANXA3
BGLAP
COL1A1
DDC
KRTAP4-3
NR5A1
PDCD4
PITX3
*** p<0.001
** p<0.01
* p<0.05
Correlation
0.2
0.1
0.0
−0.1
−0.2
−0.3
B
AMI
T cells
CD8 T cells
Cytotoxic lymphocytes
B lineage
NK cells
Monocytic lineage
Myeloid dendritic cells
Neutrophils
Endothelial cells
Fibroblasts
1
0.8
0.6
0.4
0.2
0
−0.2
−0.4
−0.6
−0.8
−1
C
403 TCGA patients
C1
C2
C3
C4
groups
subtype group (n=403)
C1 (n=35, 9%)
C2 (n=17, 4%)
C3 (n=306, 76%)
C4 (n=45, 11%)
P−value
AMI−low (n=220)
15(7%)
3(1%)
174(79%)
28(13%)
0.005
AMI−high (n=183)
20(11%)
14(8%)
132(72%)
17(9%)
AMI−low
AMI−high
D
H−TMB
L−TMB
Survival probability
p=0.006
Time(years)
E
Low−AMI
High−AMI
1.7e−05
Tumor mutation burden
F
G
MSI
MSS
MSI−L
MSI−H
0.35
0.00015
0.0027
AMI

Fig. S11 the relationships between the AME signature and tumor immunity. (A) The correlation between each TME infiltration cell type and eight key AME regulators using spearman analyses. Negative correlation was marked with blue and positive correlation with red.(*P < 0.05; **P < 0.01) (B) Correlations between AMI and the known immune cells using Spearman analysis. The negative correlation was marked with blue and positive correlation with red. (C) The proportion of immune molecular subtypes in high and low AMI group by the median AMI cut off. (D) Survival analyses for patients receiving anti-PD-L1 immunotherapy stratified by both AMI and TMB using Kaplan-Meier curves. H, high; L, Low; TMB, tumor mutation burden (P < 0.001, Log-rank test). (E) Comparison of AMI level between high and low TMB. (F-G) The survival curves of three MSI patterns were estimated by the Kaplan-Meier plotter. (P = 0.0073, Log-rank test); Comparison of AMI level across three MSI patterns.

Figure S12

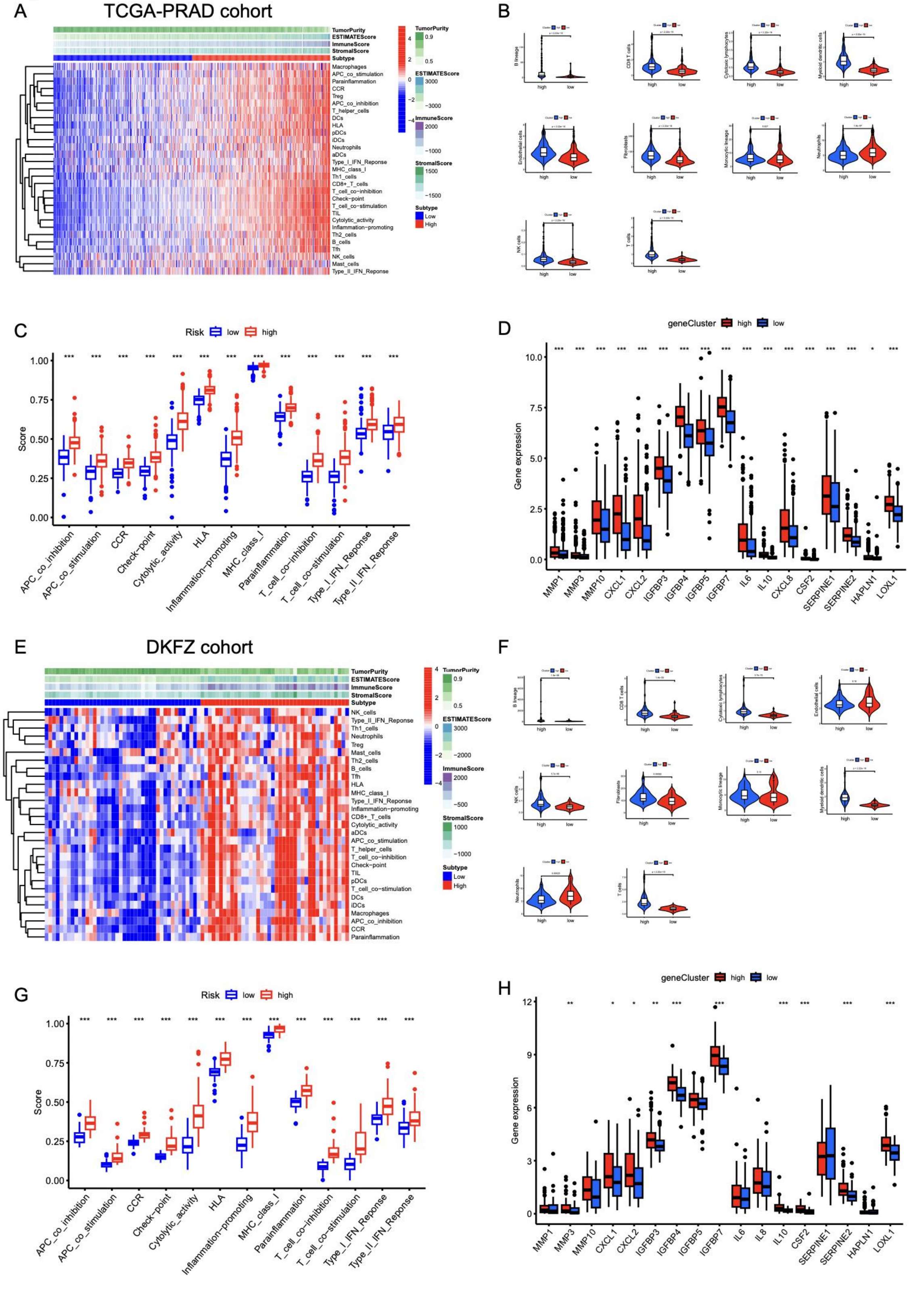

Fig. S12 The relationship between aging microenvironment and AMI cluster in

TCGA-PRAD cohort and DKFZ cohort. (A,E) The distribution of ESTIMATE score in PCa patients and heatmaps of immune score using MCP counter. (B,F) The different distribution of immune infiltrated cells between AMI-high and AMI-low subgroup. (C,G) Boxplot showing differences in the immune function between three distinct AMI clusters. (D,H) Boxplot showing differences in the expression levels of senescence related cytokines/inflammatory factors between three distinct AMI clusters.

Figure S13

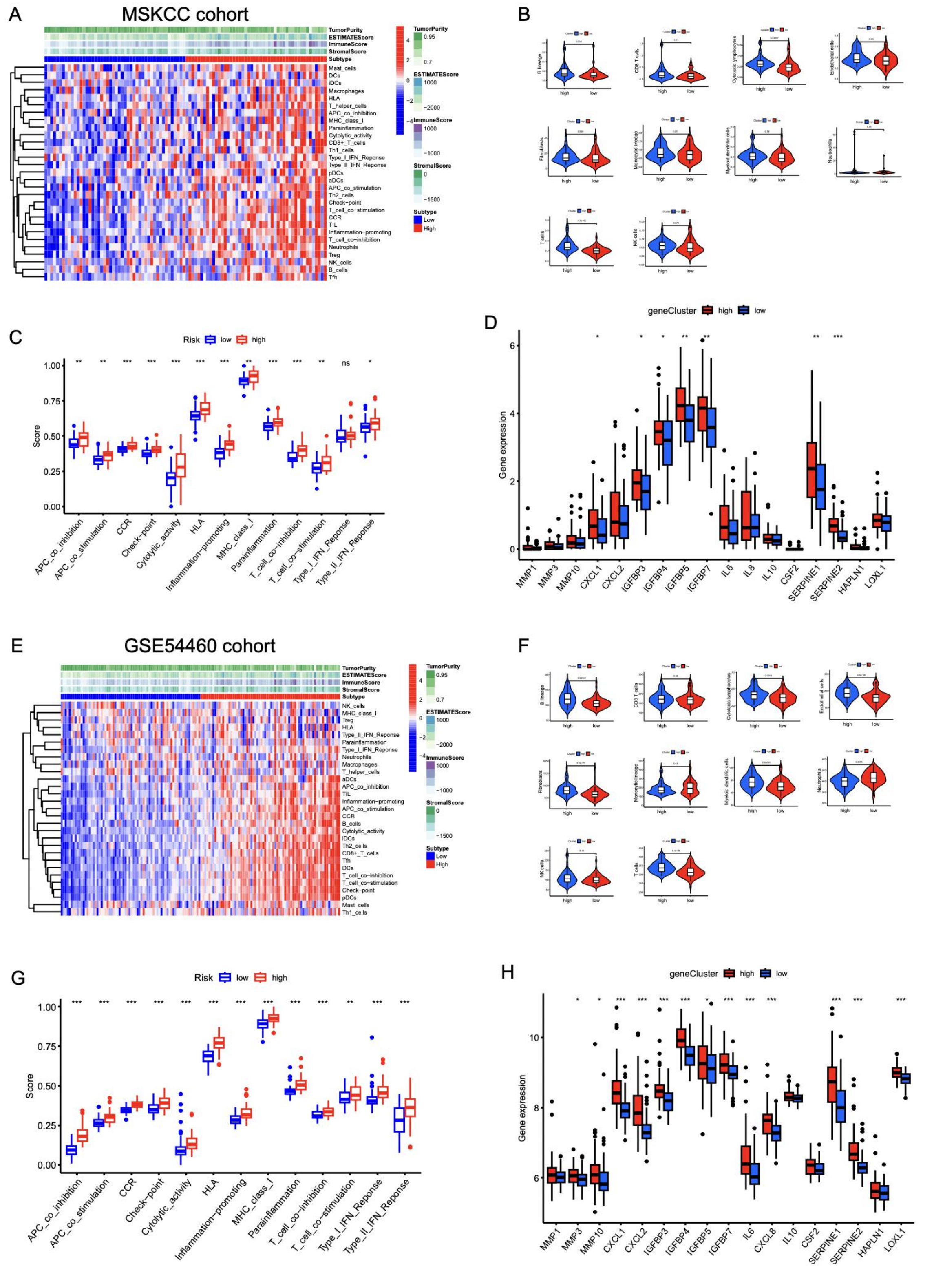

Fig. S13 The relationship between aging microenvironment and AMI cluster in MSKCC

cohort and GSE54460 cohort. (A,E) The distribution of ESTIMATE score in PCa patients and heatmaps of immune score using MCP counter. (B,F) The different distribution of immune infiltrated cells between AMI-high and AMI-low subgroup. (C,G) Boxplot showing differences in the immune function between three distinct AMI clusters. (D,H) Boxplot showing differences in the expression levels of senescence related cytokines/inflammatory factors between three distinct AMI clusters.

Figure S14

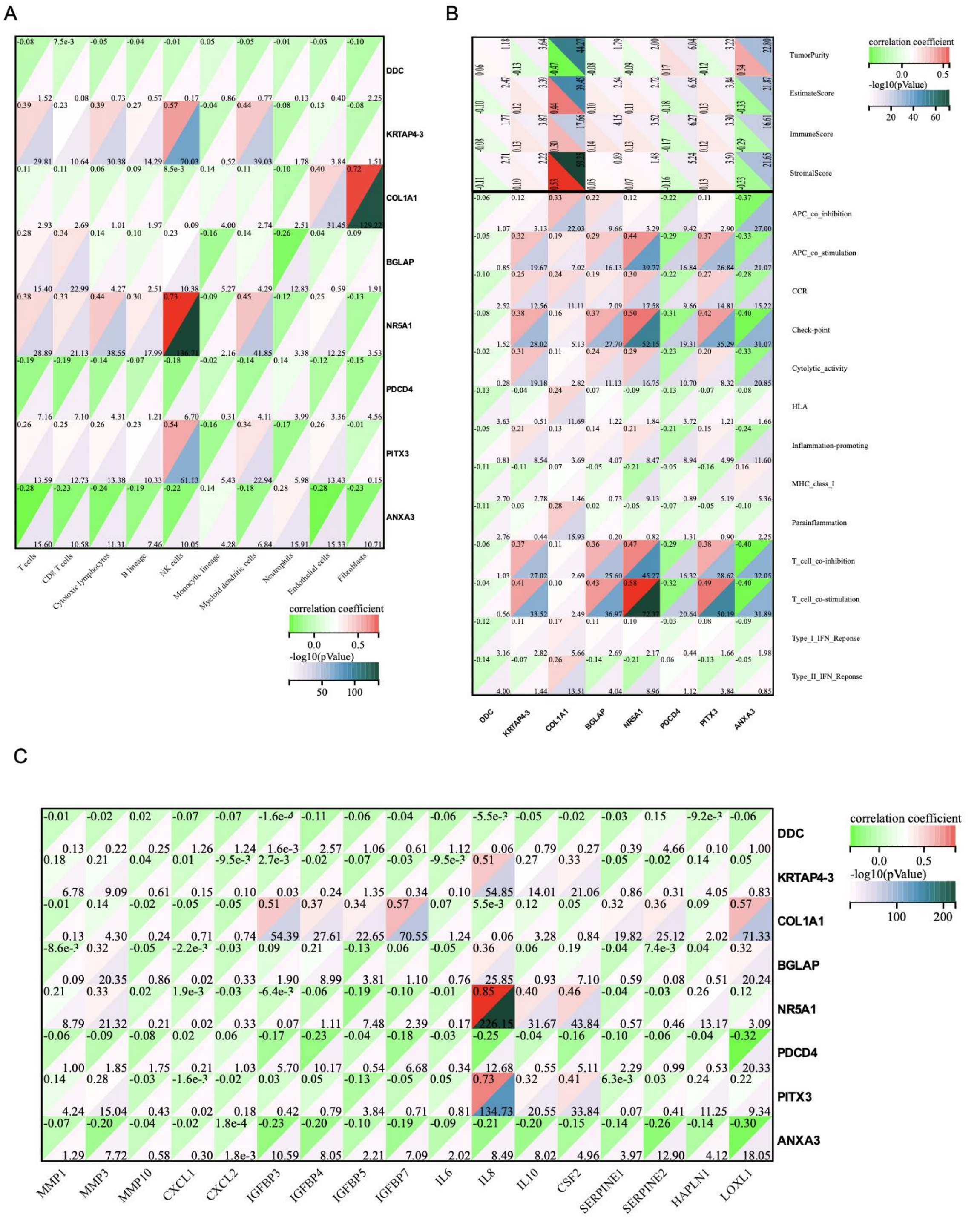

Fig. S14 (A) Correlation between immune infiltration and ARGs. (B) Correlation between TME estimate score, immune function score and ARGs. (C) Correlation between the expression levels of ageing related chemokine/cytokine/inflammatory factor

and ARGs. (for all pictures, positive correlation is represented by red, while negative correlation is represented by green. ARGs, ageing-related genes.)

FigureS15

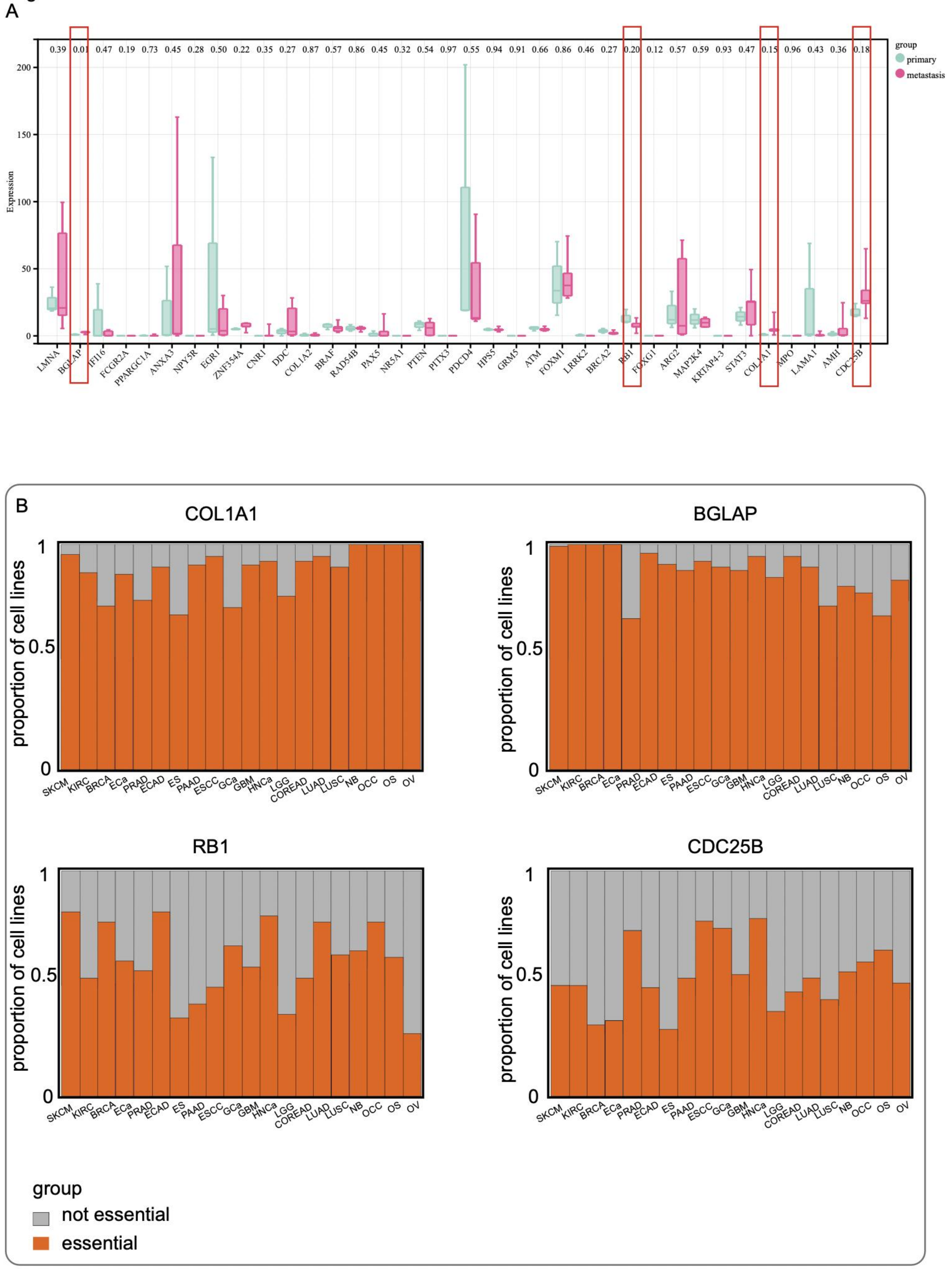
A
0.39 0.01 0.47 0.19 0.73 0.45 0.28 0.50 0.22 0.35 0.27 0.87 0.57 0.86 0.45 0.32 0.54 0.97 0.55 0.94 0.91 0.66 0.86 0.46 0.27 0.20 0.12 0.57 0.59 0.93 0.47 0.15 0.96 0.43 0.36 0.18
group
primary
metastasis
Expression
200
150
100
50
0
LMNA BGLAP IFI16 FCGR2A PPARGC1A ANXA3 NPY5R EGR1 ZNF354A CNR1 DDC COL1A2 BRAF RAD54B PAX5 NR5A1 PTEN PITX3 PDCD4 HPS5 GRM5 ATM FOXM1 LRRK2 BRCA2 RB1 FOXG1 ARG2 MAP2K4 KRTAP4-3 STAT3 COL1A1 MPO LAMA1 AMH CDC25B
B
COL1A1
BGLAP
RB1
CDC25B
proportion of cell lines
1
0.5
0
SKCM KIRC BRCA ECa PRAD ECAD ES PAAD ESCC GCa GBM HNCa LGG COREAD LUAD LUSC NB OCC OS OV
group
not essential
essential

Fig. S15 (A) The expression pattern of 36 AME regulators between 3 primary- and 5 metastasis-derived prostate cancer cell lines from CCLE. (B) The essential gene proportion of COL1A1, BGLAP, RB1, and CDC25B in pan-cancer cell lines. SKCM, Melanoma; KIRC, Kidney Carcinoma; BRCA, Breast Carcinoma; ECa, Endometrial Carcinoma; PRAD, Prostate Carcinoma; ECAD, Esophageal Adenocarcinoma; ES, Ewing`s Sarcoma; PAAD, Pancreatic Carcinoma; ESCC, Esophageal Squamous Cell Carcinoma; GCa, Gastric Carcinoma; GBM, Glioblastoma; HNCa, Head and Neck Carcinoma; LGG, Low Grade Glioma; COREAD, Colorectal Carcinoma; LUAD, Lung Adenocarcinoma; LUSC, Squamous Cell Lung Carcinoma; NB, Neuroblastoma; OCC, Oral Cavity Carcinoma; OS; Osteosarcoma; OV, Ovarian Carcinoma.

FigureS16

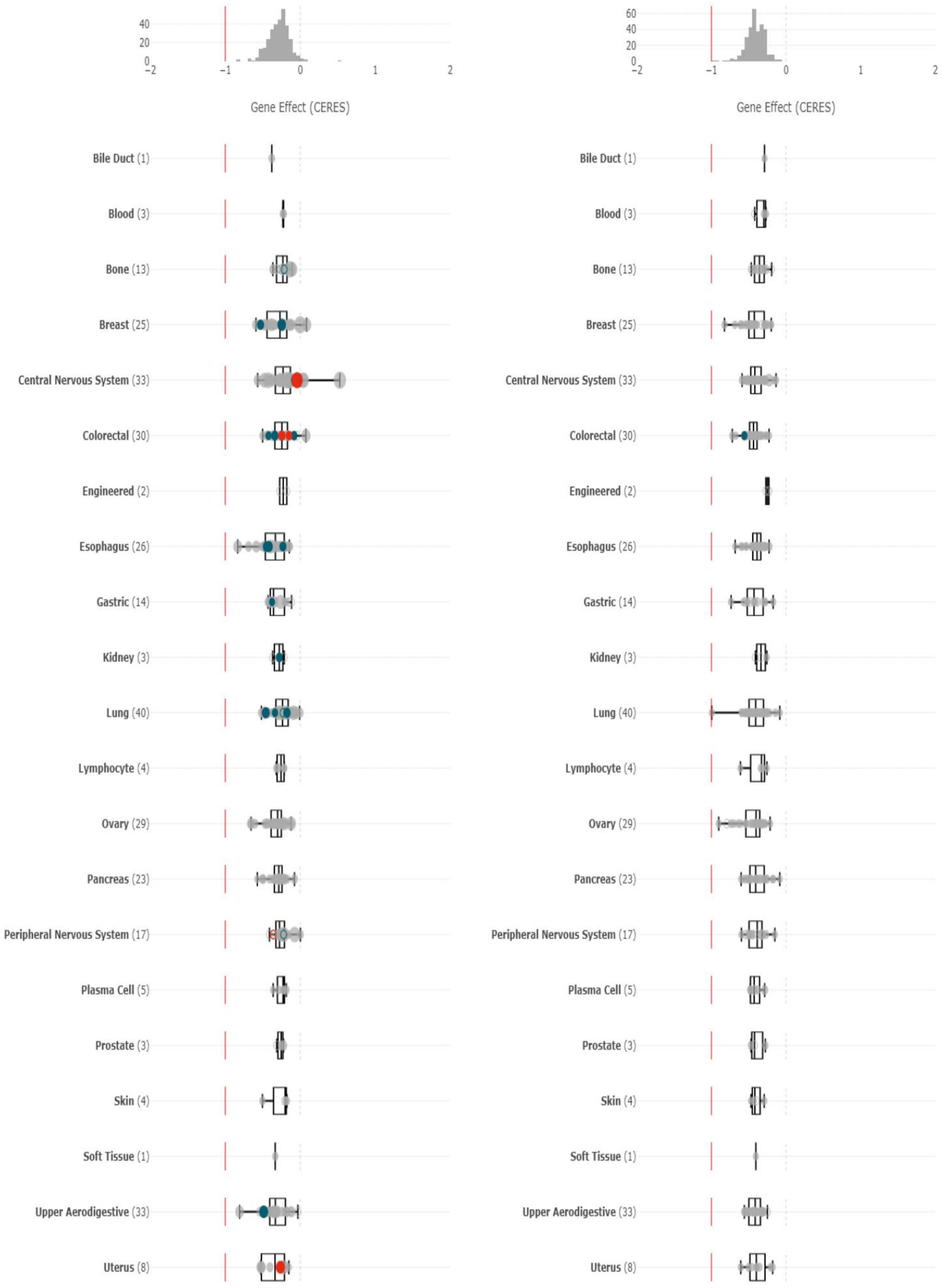

Fig. S16 The landscape of therapeutic potential of COL1A1 and BGLAP in pan-cancer

cell lines from the DepMap Portal. All the Gene Effect values are less than 0, indicating that COL1A1 and BGLAP are pro-tumoral factors in pan-cancer cells.

Figure S17

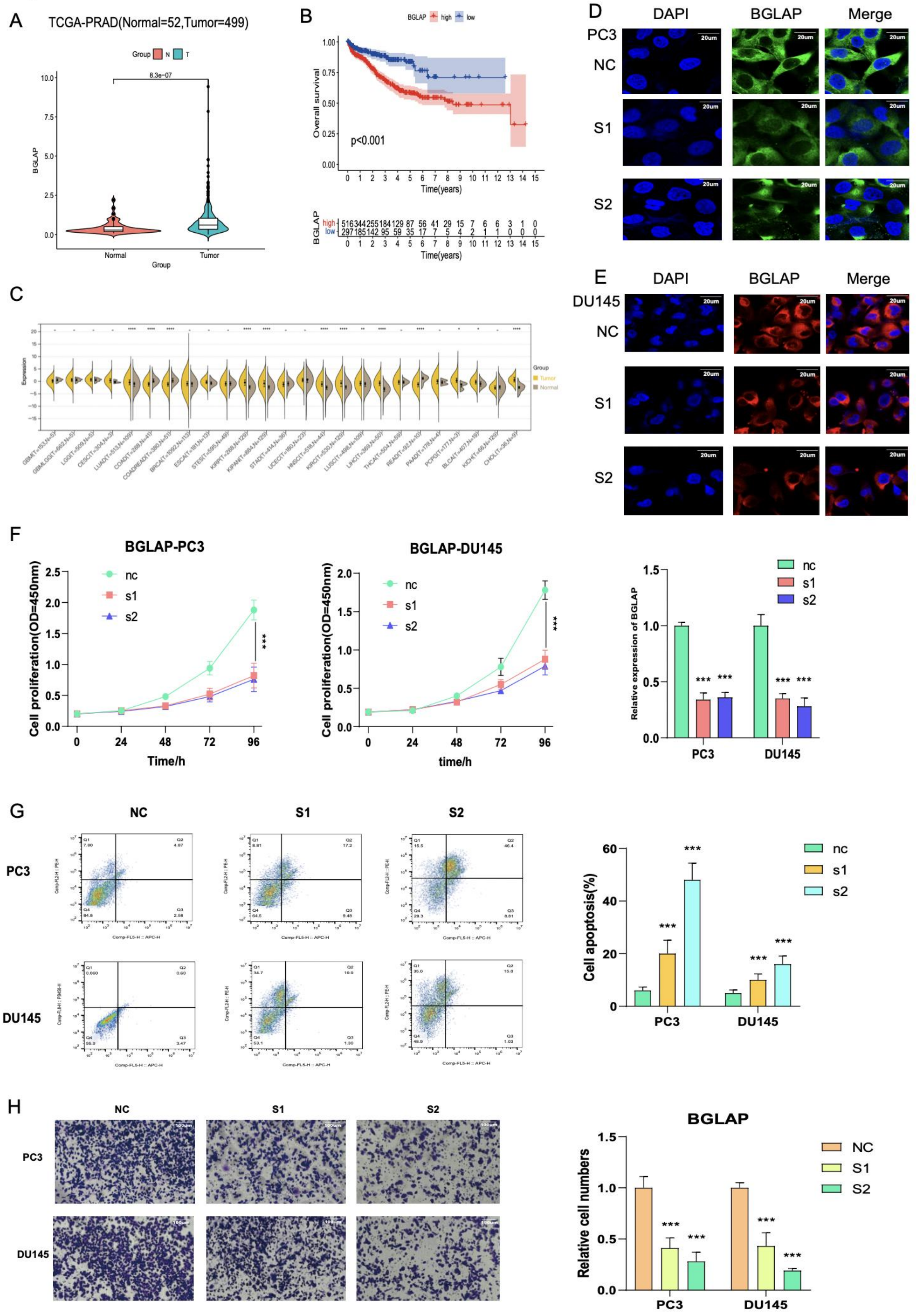

Fig. S17 BGLAP are upregulated in prostate cancer and promotes prostate cancer

progression, BGLAP positively related to PCa ISUP grading were verified in SYSU cohort by Immunohistochemistry (IHC) H score. (A) Compared with normal prostate tissue, BGLAP is upregulated in prostate cancer. (B) The survival curves of BGLAP expression was estimated by the Kaplan-Meier plotter. (P <0.001, Log-rank test). (C) The relative expression of BGLAP between tumor and normal tisuue across pan-caners. (D-E) Immunofluorescence microscopy analysis shows the expression of BGLAP in control and knockdown cells (PC3 and DU145, Scale bar, 20μm); Results of qPCR the knockdown (KD) efficiency of BGLAP. (F) The cell growth rate is evaluated in BGLAP-KD and control cells. (G) Apoptosis is determined in BGLAP-KD and control cells. (H) Transwell migration assays of the migration ability of prostate cancer cells (PC3 and DU145, magnification, Scale bar, 100μm) in the control or knockdown groups.

Figure S18

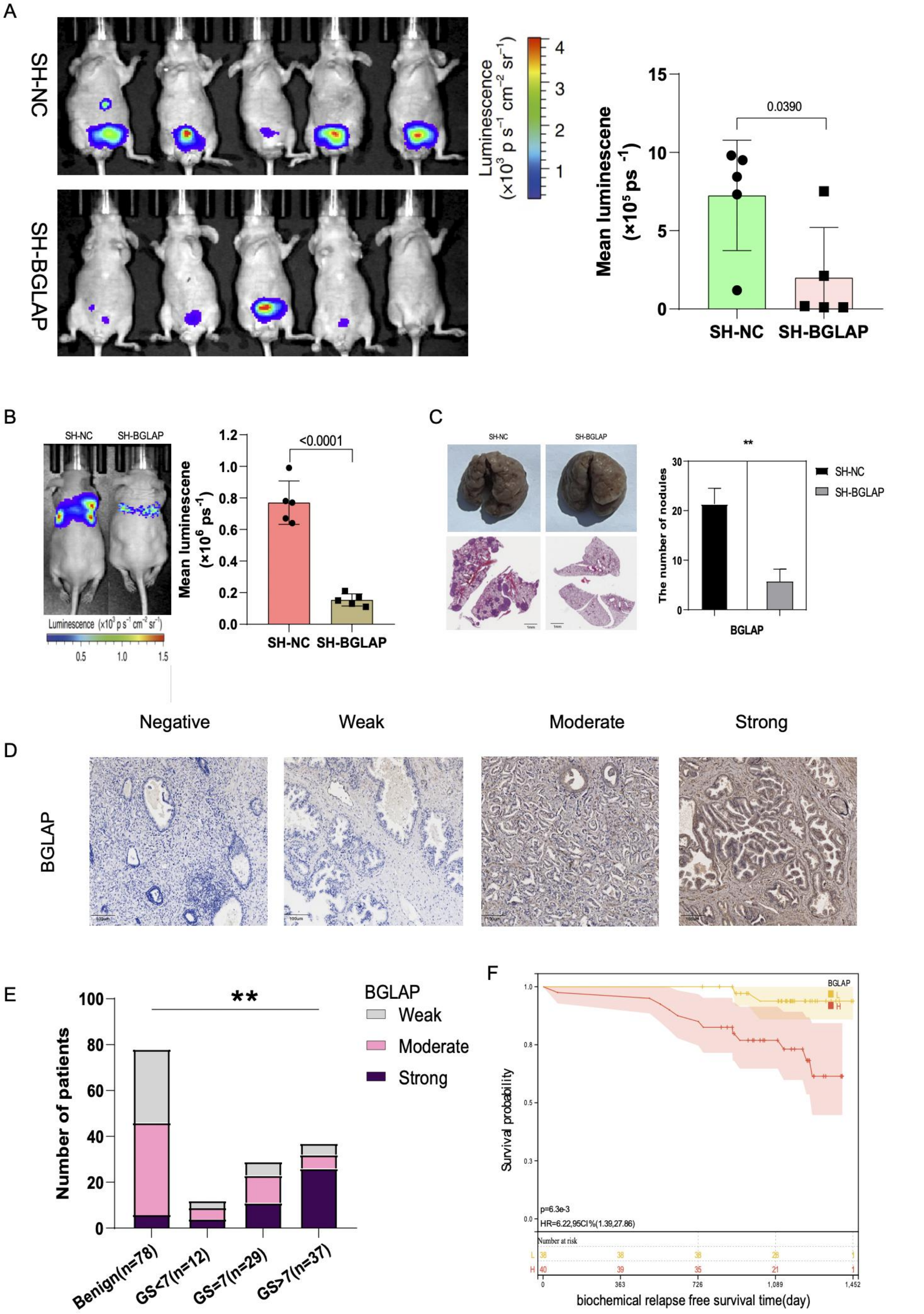

Fig. S18 (A) Orthotopic-xenograft prostate-tumor mouse models implanted with

BGLAP-KD PC3 cells. Representative bioluminescent images of orthotopic prostate tumors. Statistical calculation of the mean luminescence of the orthotopic xenograft tumors. (B) Bioluminescence of the lung metastatic nodules was detected by an in vivo bioluminescence imaging system. (C) Representative images of isolated lung tissues from the BGLAP_NC group and the BGLAP_SH group. Representative images of hematoxylin–eosin staining of lung slice from BGLAP_NC group and the BGLAP_SH group, Scale bar, 1mm. The number of metastatic nodules in the lungs from different groups. *, **, and *** represent $P<0.05$, $P<0.01$, and $P<0.001$, respectively. (D-E) The protein expression of BGLAP in different Gleason score subtypes in PCa tissues by IHC, magnification, Scale bar, 100μm. (F) The survival curves of BGLAP H score with were estimated by the Kaplan-Meier plotter. ($P = 0.0073$, Log-rank test). Comparison of bRFS between patients with a high H score and patients with a low H score was undertaken using the median value of the H score as the cutoff.

Figure S19

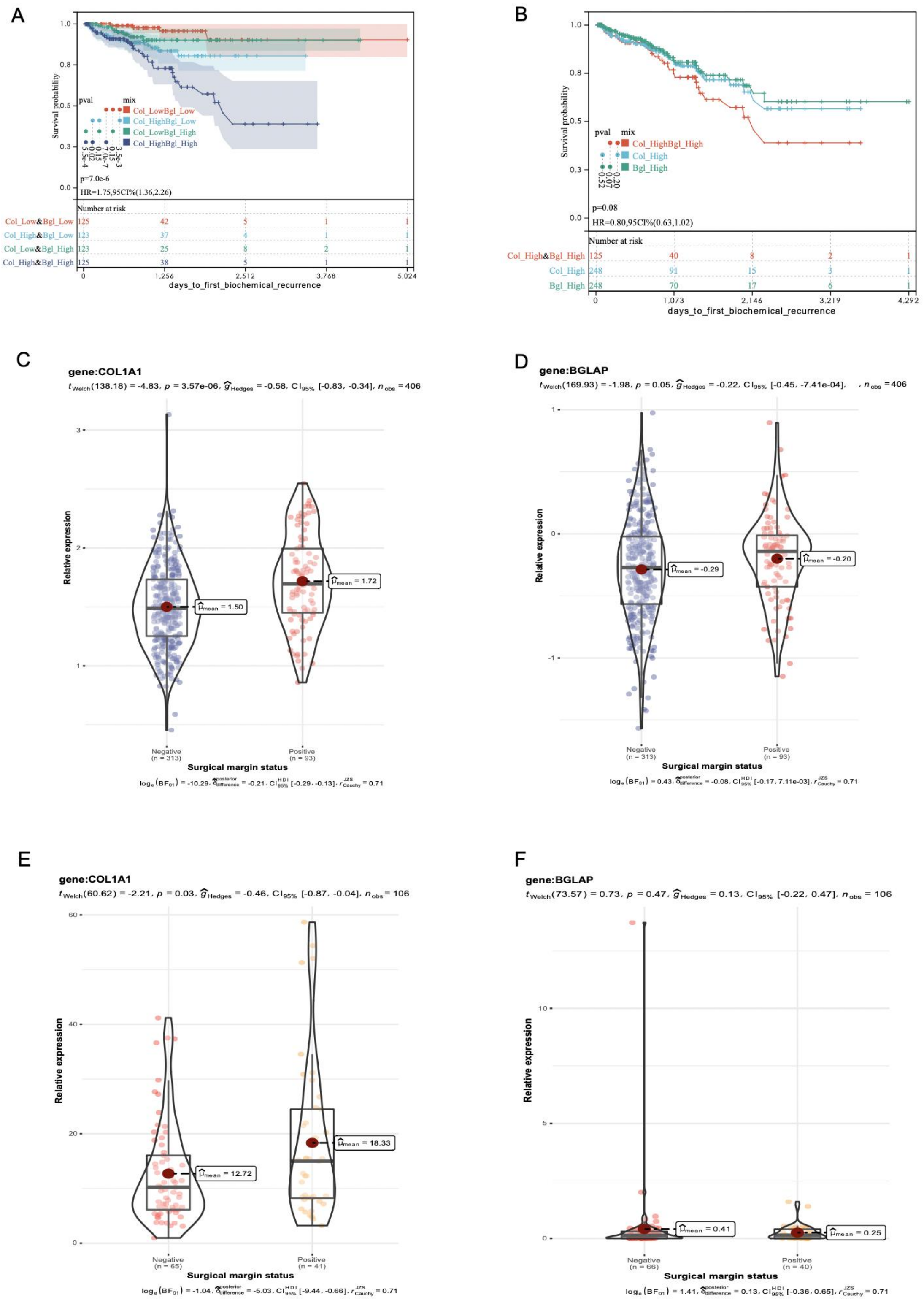

Fig. S19 (A-B) The survival curves of high COL1A1 and high BGLAP expression was estimated by the Kaplan-Meier plotter in TCGA-PRAD cohort. (Log-rank test). (C-D)

correlation between the rate of positive surgical margins and relative expression levels of COL1A1 (C) and BGLAP (D) in TCGA-PRAD cohort. (E-F) correlation between the rate of positive surgical margins and relative expression levels of COL1A1 (E) and BGLAP (F) in GSE54460 cohort.

Supplementary Tables:

Table S1. Ageing related genes

Ageing related genes										
ANXA3	AC037482.3	BCL6	CREB1	FOXM1	IGFBP5	LONP1	MORC3	PCK1	RETN	TERF2
FCGR2A	ADA	BECN1	CRYAB	FOXO4	IL10	LOXL2	MPO	PDCD4	RGN	TERT
STAT3	ADM	BGLAP	CTC1	FZR1	IL15	LRP1	MSH2	PDGFRB	RNF165	TFCP2L1
RB1	ADRA1A	BMPR1A	CTNNA1	GBA	ING2	LRRK2	MSH6	PDX1	ROMO1	TGFB3
MAP2K4	AGER	BRCA2	CTSC	GCLM	INPP5D	MAGEA2	MT-ATP6	PENK	RPN2	TGFBR2
LAMA1	AKT1	C1QA	CYP1A1	GHRHR	IRAK1	MAGEA2B	MT-CO1	PICALM	RPS6KB1	TH
HPS5	AKT3	CACYBP	DAG1	GJB2	ITGB2	MAP2K1	MT-ND4	PITX3	RSL1D1	TIMP1
EGR1	ALDH3A1	CALCA	DCN	GJB6	JUN	MAP3K3	MTOR	PLA2R1	SCAP	TIMP2
CDC25B	ALOX12	CALR	DDC	GLRX2	JUND	MAPK1	NAPEPLD	PLK2	SEC63	TNFRSF1B
IFI16	AMFR	CARM1	DKK1	GNAO1	KAT6A	MAPK14	NEK4	PML	SERPINE1	TP53
COL1A2	AMH	CASP2	DLD	GNRH1	KCNE2	MAPK3	NEK6	PNPT1	SERPINF1	TP63
BRAF	APAF1	CAT	DNAJA3	GRB2	KCNMB1	MAPKAPK5	NFE2L2	POLB	SIN3A	
ZNF354A	APEX1	CCL11	DNMBP	GRM5	KIR2DL4	MARCHF5	NFKB2	POLG	SIRT1	
ACSS2	APOD	CCN2	DNMT3A	GSK3A	KL	MBD2	NOX4	PPARGC1A	SIRT3	
ATP5MC3	APP	CD68	ECRG4	GSN	KMO	MBD3	NPM1	PPP1R9A	SLC12A2	
CA4	ARG1	CDK6	EDN1	GSS	KRAS	MIF	NPY2R	PPP1R9B	SLC30A10	
CALB1	ARG2	CDKN1A	EDNRB	H2AX	KRT14	MIR10A	NPY5R	PPP3CA	SLC32A1	
COL1A1	ARNTL	CDKN2A	EEF1E1	HAMP	KRT16	MIR146A	NQO1	PRDM2	SLC6A3	
COL3A1	ASS1	CDKN2B	EEF2	HLA-G	KRT25	MIR17	NR5A1	PRELP	SMC5	
COL4A5	ATG7	CGAS	EIF2S1	HMGA1	KRT33B	MIR188	NSMCE2	PRKCD	SMC6	
CX3CL1	ATM	CHEK1	ENDOG	HMGA2	KRT83	MIR20B	NTRK1	PRKDC	SOD1	
DIABLO	ATP2B1	CHEK2	ENO3	HRAS	KRTAP4-3	MIR21	NUAK1	PRMT6	SOD2	
FABP3	ATP8A2	CISD2	EPO	HTR2A	KRTAP4-5	MIR217	NUDT1	PRNP	SPI1	
GHITM	ATR	CLDN1	ERCC1	HTRA2	KRTAP4-8	MIR22	NUP62	PSEN1	SREBF1	
NDUFB11	AURKB	CLN8	ERCC2	HYAL2	KRTAP4-9	MIR34A	OGG1	PTEN	SRF	
NREP	B2M	CNP	ERO1A	ICAM1	KYNU	MIR543	OPA1	PTH1R	SRR	
TFRC	BAK1	CNR1	FBXO4	ID2	LEP	MIR590	P2RY1	RAD54B	TACR3	
UQCRFS1	BCL2	COL4A2	FBXO5	IDE	LIMS1	MME	PAWR	RAD54L	TBX2	
UQCRQ	BCL2A1	COMP	FOS	IGFBP1	LITAF	MMP7	PAX2	RBL1	TBX3	
ABL1	BCL2L12	COQ7	FOXG1	IGFBP2	LMNA	MNT	PAX5	RELA	TERC	

Table S2. Univariate cox regression analyses of 18 bRFS-positive regulators and 18 bRFS-negative regulators.

id	HR	HR.95L	HR.95H	pvalue	km
NPY5R	1.809412475	0.85803924	3.815645429	0.119288147	0.036182921
MPO	0.882088067	0.268091578	2.902289449	0.836420178	0.128537886
COL1A1	1.003103643	1.002093079	1.004115227	1.68E-09	6.11E-15
STAT3	0.977556267	0.958639679	0.996846132	0.022797931	8.43E-05
RB1	0.924914865	0.870053663	0.983235338	0.012353438	0.000690692
RAD54B	1.515246128	0.980377381	2.341925542	0.061379295	0.00704284
PPARGC1A	0.301973032	0.185886406	0.490556108	1.32E-06	2.82E-06
MAP2K4	0.883773107	0.825702416	0.945927841	0.000366629	8.55E-07
PAX5	1.109994098	0.958850764	1.284962107	0.162318615	0.089784509
LMNA	0.999176349	0.990286859	1.008145637	0.856590544	0.05785237
LAMA1	1.248056164	0.949499674	1.640489441	0.112177061	0.00660233
HPS5	0.771975996	0.625514741	0.952730447	0.015904797	0.002576576
GRM5	3.542178435	0.788410114	15.91434185	0.098975802	0.066102457
FOXM1	1.138242096	1.077048644	1.202912308	4.38E-06	7.72E-11
FOXG1	1.52842819	1.26054777	1.853236179	1.60E-05	0.197965389
EGR1	0.997460279	0.996177383	0.998744826	0.000107647	5.83E-10
CDC25B	1.140266188	1.068196636	1.21719816	8.13E-05	3.63E-05
LRRK2	0.825955935	0.527101194	1.294254717	0.404052549	0.000290747
IFI16	1.006186577	0.95953043	1.05511133	0.799031733	0.007280565
COL1A2	1.004824363	1.003013474	1.006638522	1.70E-07	1.97E-07
CNR1	2.860977283	1.329615506	6.156058633	0.007174131	0.007248982
BRAF	0.982243562	0.898598634	1.073674473	0.693186628	0.010848466
BRCA2	2.625238808	1.342059305	5.13530123	0.004811827	2.11E-07
PTEN	0.888889655	0.837970521	0.942902881	9.10E-05	7.53E-07
ATM	0.891995769	0.747468864	1.064467686	0.205062429	0.005940172
AMH	1.098403487	1.055667481	1.142869551	3.56E-06	7.88E-09
ARG2	0.991512463	0.986418092	0.996633144	0.001182102	8.90E-07
BGLAP	1.215934519	1.098437345	1.34600008	0.000162769	1.70E-06
DDC	1.038239234	1.020212194	1.056584809	2.68E-05	1.85E-05
KRTAP4-3	0.806391095	0.486559484	1.336458582	0.403820748	0.140847582
NR5A1	0.938764154	0.324648741	2.714558924	0.907144339	0.304682077
PDCD4	0.972544951	0.958978579	0.986303242	0.000102671	1.11E-08
PITX3	0.750698908	0.316171076	1.782417474	0.515727986	0.004115187
ZNF354A	1.269616535	1.088554976	1.480794431	0.002359323	0.000886006
ANXA3	0.942581238	0.920991724	0.964676844	5.68E-07	2.99E-10
FCGR2A	1.288462179	1.156206253	1.435846573	4.51E-06	1.22E-06

Table S3.CMap database was used to screen for top 30 small-molecule drugs

rank	cmap name	mean	n	enrichment	p	specificity	percent non-null
1	fludrocortisone	-0.307	8	-0.651	0.0008	0.0423	50
2	Prestwick-692	-0.297	4	-0.849	0.00097	0.0068	50
3	timolol	-0.244	4	-0.834	0.00137	0	50
4	oxetacaine	0.405	5	0.752	0.00228	0.0121	60
5	Prestwick-664	0.399	6	0.688	0.00238	0.0072	66
6	ajmaline	-0.44	3	-0.882	0.0032	0.0142	66
7	ribavirin	-0.455	4	-0.792	0.00376	0.0395	75
8	vancomycin	-0.31	4	-0.79	0.00398	0.0069	50
9	Gly-His-Lys	-0.358	3	-0.87	0.00437	0.0224	66
10	chlorhexidine	-0.316	5	-0.707	0.00469	0.015	60
11	naringenin	-0.344	4	-0.778	0.00503	0.0323	50
12	lasalocid	-0.46	4	-0.764	0.00635	0.0556	75
13	3-acetamidocoumarin	-0.313	4	-0.753	0.00758	0.1234	50
14	ikarugamycin	-0.404	3	-0.843	0.00773	0.0227	66
15	clorsulon	-0.229	4	-0.751	0.00774	0.0284	50
16	iloprost	-0.322	3	-0.834	0.00915	0.0188	66
17	azacitidine	0.46	3	0.833	0.00931	0.0865	66
18	tropicamide	0.286	6	0.606	0.0117	0.0145	50
19	thapsigargin	-0.419	3	-0.802	0.01572	0.1613	66
20	pseudopelletierine	0.381	4	0.701	0.01643	0.0184	50
21	mefloquine	0.343	5	0.64	0.01734	0.201	60
22	vorinostat	0.3	12	0.421	0.0183	0.6181	50
23	xylometazoline	0.476	4	0.69	0.01932	0.0076	75
24	indoprofen	-0.255	4	-0.69	0.01995	0.06	50
25	pargyline	0.337	4	0.686	0.02075	0.063	50
26	trifluridine	0.282	4	0.685	0.02117	0.1	50
27	MK-886	-0.393	2	-0.894	0.02276	0.0133	100
28	perhexiline	0.527	4	0.678	0.02322	0.1244	75
29	Prestwick-857	-0.403	4	-0.674	0.02522	0.0446	75
30	Prestwick-674	0.333	6	0.561	0.02666	0.0274	50

Table S4. Sequences of primer and siRNA used in this study

primers for qRT-PCR	5' to 3'	
COL1A1-F	GAGGGCCAAGACGAAGACATC	
COL1A1-R	CAGATCACGTCATCGCACAAC	
BGLAP-F	CACTCCTCGCCCTATTGGC	
BGLAP-R	CCCTCCTGCTTGGACACAAAG	
GAPDH-F	GGAGCGAGATCCCTCCAAAAT	
GAPDH-R	GGCTGTTGTCATACTTCTCATGG	
**siRNA sequences**		
**siRNAs**	**sense (5' to 3')**	**antisense (5' to 3')**
si-COL1A1#1	UGUAGUACCAGCUACUUGGGA	CCAAGUAGCUGGUACUACAGG
si-COL1A1#2	UAAAAAUACAAAAAUUAGCCC	GCUAAUUUUUGUAUUUUUAGU
si-BGLAP#1	ACAAUGUACUCCAUAUUGCAA	GCAAUAUGGAGUACAUUGUUG
si-BGLAP#2	AUAGUUAACAACAAUGUACUC	GUACAUUGUUGUUAACUAUAG
si-NC	UUCUCCGAACGUGUCACGUTT	ACGUGACACGUUCGGAGAATT

**antibody for Western blot and IHC**
COL1A1 (Abcam,ab138492;WB:1:1000, IHC:1:200)
BGLAP (Abcam,ab93876;WB:1:1000, IHC:1:200)
